# Role of the cGAS-STING signaling pathway in diabetes mellitus and its complications: from mechanisms to therapeutics

**DOI:** 10.3389/fphar.2026.1891745

**Published:** 2026-09-03

**Authors:** Guangdong Qi, Tongxin Shang, Hualin Sun, Jie Wang, Lanxiang Hao

**Affiliations:** 1 Department of Clinical Medicine, Clinical Medical College, Yangzhou University, Yangzhou, Jiangsu, China; 2 Department of Endocrinology, Binhai County People’s Hospital, Yancheng, Jiangsu, China; 3 Jiangsu Key Laboratory of Tissue Engineering and Neuroregeneration, Key Laboratory of Neuroregeneration of Ministry of Education, Co-Innovation Center of Neuroregeneration, Medical School of Nantong University, Nantong University, Nantong, Jiangsu, China; 4 Department of Endocrinology, The First People’s Hospital of Yancheng, Yancheng, Jiangsu, China

**Keywords:** cGAS-STING, diabetes mellitus, diabetic complications, inflammation, mitochondrial DNA, therapeutic strategies

## Abstract

Diabetes mellitus and its complications represent a major global public health challenge, with their pathogenesis closely linked to chronic, low-grade, non-infectious metabolic inflammation. The cGAS-STING signaling pathway, a crucial innate immune sensor of cytosolic DNA, has recently emerged as a central hub linking metabolic stress to sterile inflammation. This review systematically elucidates the mechanisms and therapeutic potential of the cGAS-STING pathway in diabetes mellitus and its associated complications. Under diabetic metabolic stress conditions—such as hyperglycemia and lipotoxicity—mitochondrial dysfunction and nuclear DNA damage lead to the leakage of DNA into the cytoplasm, which acts as damage-associated molecular patterns (DAMPs) to activate the cGAS-STING pathway. Aberrant activation of this pathway is extensively involved in the pathogenesis of various diabetic complications, including diabetic cardiomyopathy (DCM), nephropathy, retinopathy, foot ulcers, and macrovascular disease, driving tissue damage through mechanisms such as pyroptosis, inflammatory responses, fibrosis, and cellular senescence. Furthermore, the cGAS-STING signaling cascade plays a critical role in core pathological processes of diabetes mellitus, including the regulation of insulin resistance, adipose tissue inflammation, and pancreatic β-cell dysfunction. Current intervention strategies targeting this pathway—comprising small molecule inhibitors (e.g., STING inhibitors C-176/H-151, cGAS inhibitor RU.521), strategies for mitochondrial quality control (e.g., modulating mitophagy and mitochondrial dynamics), and various natural products and traditional Chinese medicine formulations—have demonstrated significant therapeutic promise in preclinical models. Nevertheless, challenges remain in this field, including cell-type specificity, therapeutic windows, pathway redundancy, and a lack of reliable biomarkers. Future directions aimed at advancing targeted therapies of the cGAS-STING pathway from bench to bedside should focus on leveraging single-cell multi-omics technologies to decipher its spatiotemporal specificity, developing precision-targeted delivery systems, exploring multi-target combination strategies, and establishing clinically applicable biomarkers. This review provides a novel perspective on the inflammatory mechanisms underlying diabetes mellitus and its complications, thereby establishing a theoretical foundation for the development of therapeutic strategies centered on modulating the cGAS-STING pathway.

## Graphical Abstract

**Figure:**
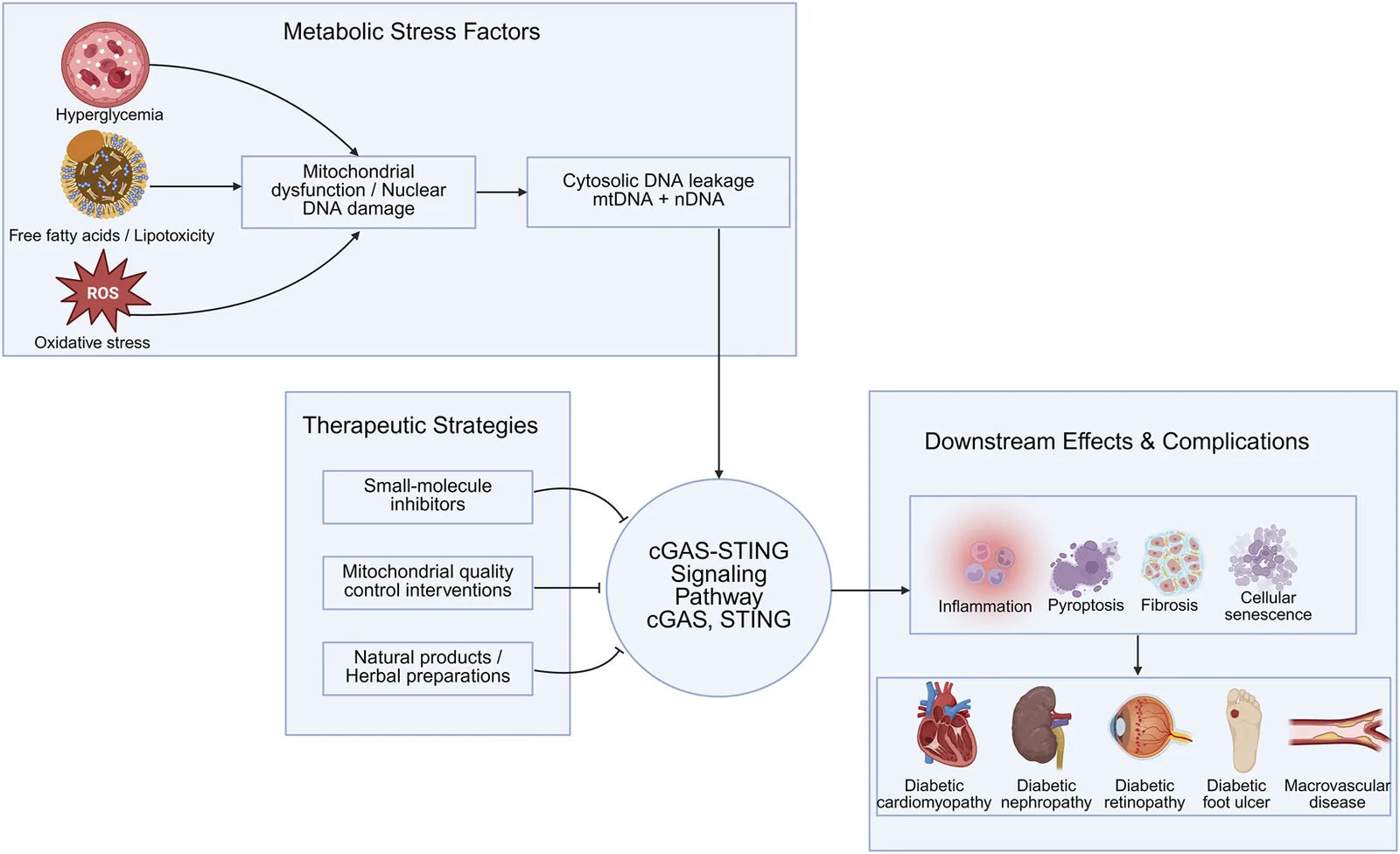
Metabolic stress (hyperglycemia, free fatty acids, and reactive oxygen species) induces mitochondrial dysfunction and nuclear DNA damage, leading to the cytosolic leakage of mitochondrial DNA (mtDNA) and double-stranded DNA (dsDNA). This event activates the cGAS-STING pathway, triggering downstream inflammatory responses, pyroptosis, fibrosis, and cellular senescence. These pathological processes collectively contribute to major diabetic complications, including diabetic cardiomyopathy, nephropathy, retinopathy, foot ulcers, and macrovascular disease. Current therapeutic strategies targeting this pathway include small-molecule inhibitors, mitochondrial quality control interventions, and natural products. Created in BioRender. (2026) https://BioRender.com/t4aep68.

## 1 Introduction

Diabetes Mellitus (DM) is a chronic metabolic disorder characterized by hyperglycemia, and its continuously escalating global prevalence has established it as one of the most critical public health challenges of the 21st century (Gong et al., 2025; Yi et al., 2026). Based on etiology and clinical presentation, DM is primarily classified into type 1 diabetes mellitus (T1DM), resulting from autoimmune-mediated destruction of pancreatic β-cells, and type 2 diabetes mellitus (T2DM), characterized by insulin resistance and progressive β-cell dysfunction (Mohammadi and Khorasani, 2024). The true burden of diabetes mellitus, however, stems from its long-term complications, which are broadly categorized into microvascular (e.g., diabetic kidney disease (DKD), diabetic retinopathy (DR)) and macrovascular complications (e.g., cardiovascular disease, cerebrovascular disease, peripheral arterial disease) (Sun et al., 2026; Sun et al., 2025a; Deng et al., 2024). These complications represent the predominant causes of disability and mortality among diabetic patients. Consequently, a deep exploration of the pathogenic mechanisms underlying diabetes mellitus and its complications holds significant clinical importance.

Chronic, low-grade, non-infectious inflammation has long been recognized as a common pathological basis for the onset and progression of diabetes mellitus and its associated complications. This persistent inflammatory state, termed ‘inflammaging,’ is now understood to be a key driver of age-related metabolic dysfunction, with the cGAS-STING pathway emerging as a central mediator of this sterile inflammation (Wei et al., 2026). In the context of obesity and insulin resistance, inflammatory responses—involving both metabolic cells (such as adipocytes, hepatocytes, and myocytes) and immune cells—constitute a critical bridge that links metabolic dysregulation to tissue damage. Although various pattern recognition receptors, including Toll-like receptors (TLRs) and NOD-like receptors (NLRs), have been implicated in regulating metabolic inflammation, recent research has unveiled a more central inflammatory axis: the cGAS-STING signaling pathway (He et al., 2024a). Consequently, elucidating the mechanistic role of this pathway in metabolic inflammation has become a Frontier focus in current diabetes research. Of particular relevance, the cGAS-STING pathway has been shown to critically dictate macrophage polarization between the pro-inflammatory M1 and the reparative M2 phenotypes, thereby exerting profound effects on the inflammatory microenvironment in metabolic disorders (Liu et al., 2026). Collectively, these insights not only deepen our understanding of diabetes pathogenesis but also highlight the cGAS-STING axis as a promising therapeutic target for mitigating inflammation-driven metabolic complications.

The cGAS-STING pathway serves as a pivotal innate immune defense mechanism for sensing aberrant cytoplasmic DNA, such as viral DNA or self-genomic DNA (Liu et al., 2026; Jiang et al., 2026). Under physiological conditions, DNA is strictly segregated within the nucleus and mitochondria. However, under pathological circumstances, the appearance of “non-self” DNA in the cytoplasm is recognized by cyclic GMP-AMP synthase (cGAS), which catalyzes the synthesis of the second messenger 2′3′-cyclic GMP-AMP (cGAMP). cGAMP subsequently binds to stimulator of interferon genes (STING) located on the endoplasmic reticulum membrane, activating STING and prompting its translocation to the Golgi apparatus. This leads to the recruitment and phosphorylation of TANK-binding kinase 1 (TBK1) and interferon regulatory factor 3 (IRF3), ultimately inducing the production of type I interferons (IFN-I) and a range of pro-inflammatory cytokines (He et al., 2024a). Furthermore, STING can also activate the nuclear factor-kappa B (NF-κB) pathway, thereby further amplifying the inflammatory response (Mohammadi and Khorasani, 2024). This complex signaling cascade intimately links cellular stress conditions with inflammatory outcomes.

In the context of diabetes mellitus and its complications, a spectrum of metabolic stressors—including hyperglycemia, elevated free fatty acids, oxidative stress, and endoplasmic reticulum stress—has been shown to induce mitochondrial dysfunction (He et al., 2024a). Mitochondria, functioning as the cell’s powerhouse and a primary source of reactive oxygen species (ROS), undergo dysfunction that can directly lead to the loss of mitochondrial membrane potential and the opening of the mitochondrial permeability transition pore (mPTP), consequently resulting in the leakage of mitochondrial DNA (mtDNA) into the cytoplasm (Ma et al., 2023a). Owing to its abundance of unmethylated CpG motifs—a structure resembling bacterial DNA—mtDNA is recognized by cGAS as a damage-associated molecular pattern (DAMP), thus activating the cGAS-STING pathway. This “mitochondria-inflammation” axis has emerged as a key mechanism linking metabolic stress to sterile inflammation (He et al., 2024a; Ma et al., 2023a), providing a novel perspective for understanding the pathogenesis of diabetic complications.

Although earlier reviews have established the foundational role of the cGAS-STING pathway in diabetes (Mohammadi and Khorasani, 2024; He et al., 2024a), the rapid accumulation of new findings since 2024 warrants a timely update. The present review is distinguished from prior publications by integrating these recent discoveries into a comprehensive and updated framework. First, we provide a systematic analysis of the spatiotemporal and cell-type-specific functions of the cGAS-STING pathway across diabetic complications, highlighting not only its pathogenic roles but also context-dependent protective functions. Second, we describe the emerging regulatory layers, including epitranscriptomic RNA methylation, mitochondrial quality control checkpoints, and endoplasmic reticulum–mitochondria contact sites. Third, we critically evaluate the latest therapeutic advancements—from small-molecule inhibitors and natural products to PROTACs, cell-type-specific nanocarriers, and mitochondrial-targeted interventions—with particular emphasis on translational barriers and future clinical prospects.

This review aims to comprehensively elucidate the mechanistic roles of the cGAS-STING signaling pathway in diabetes mellitus and its various complications. We will first revisit the mechanisms by which this pathway is activated under diabetes mellitus-associated metabolic stress. Subsequently, we will systematically discuss its pathological involvement in major diabetic complications, including DCM, nephropathy, retinopathy, foot ulcers, macrovascular disease, and neuropathy (He et al., 2024a). Following this, we will explore the potential regulatory functions of this pathway in insulin resistance and pancreatic β-cell function. Finally, we will summarize current therapeutic strategies targeting this pathway, analyze the challenges associated with their clinical translation, and propose future research directions, aiming to provide novel targets and insights for the prevention and treatment of diabetes mellitus and its complications.

## 2 Mechanisms of cGAS-STING pathway activation under metabolic stress in diabetes mellitus

**FIGURE 1:**
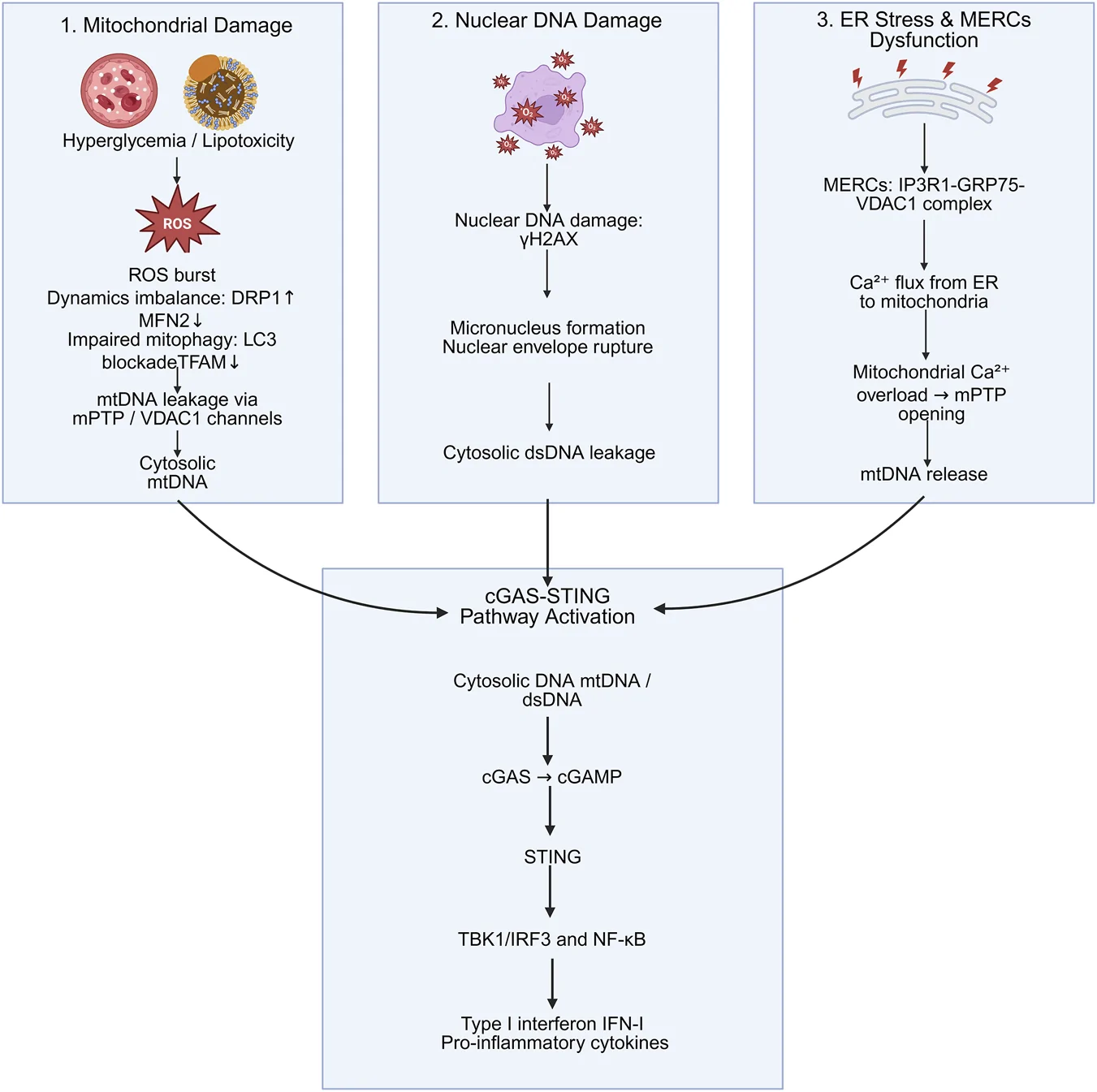
Mechanisms of cytosolic DNA leakage under diabetic metabolic stress. Hyperglycemia and lipotoxicity induce mitochondrial damage through excessive reactive oxygen species production, imbalance of mitochondrial dynamics (upregulation of DRP1 and downregulation of MFN2), impaired mitophagy (e.g., reduced TFAM expression), and subsequent mtDNA leakage via the mitochondrial permeability transition pore and VDAC1 channels. Concurrently, oxidative stress-driven nuclear DNA damage (marked by γH2AX) leads to micronucleus formation and nuclear envelope rupture, releasing dsDNA into the cytosol. Additionally, endoplasmic reticulum stress and dysfunction of mitochondria-endoplasmic reticulum contacts alter calcium flux via the IP3R1-GRP75-VDAC1 complex, resulting in mitochondrial calcium overload and mPTP opening. These convergent events promote cytosolic accumulation of mtDNA and dsDNA, which ultimately activates the cGAS-STING signaling cascade. Created in BioRender. (2026) https://BioRender.com/jgq0zyk.

The core pathological features of diabetes mellitus are persistent hyperglycemia and dyslipidemia. These metabolic abnormalities collectively constitute a complex “metabolic stress” environment, which serves as the initiating factor for the activation of the cGAS-STING pathway. Current research has revealed at least three major mechanisms that intertwine to collectively drive the inflammatory processes underlying diabetes mellitus and its complications (Figure 1).

### 2.1 Mitochondrial damage and mtDNA leakage

Mitochondria are not only the energy factories of the cell but also the most critical “amplifiers” of the cGAS-STING pathway. Indeed, significant mitochondrial dysfunction has been observed in the heart, kidney, retina, and vascular endothelial cells of diabetic patients, and both high glucose and high free fatty acids (particularly palmitate) can directly damage mitochondria, leading to decreased membrane potential, abnormal respiratory chain complex activity, imbalanced dynamics, and impaired mitophagy (Ma et al., 2023a; Yan et al., 2022). Ultimately, these diverse injuries converge on a common final pathway—the leakage of mitochondrial DNA (mtDNA) into the cytoplasm, where it acts as a damage-associated molecular pattern (DAMP) recognized by cGAS, thereby triggering a downstream inflammatory cascade (Ma et al., 2023a; Tsvetkov et al., 2022). Moreover, the extent of mtDNA leakage appears to be positively correlated with the degree of metabolic stress and is further exacerbated by the synergistic effects of aging and long-term high-fat diet (Tsvetkov et al., 2022). Therefore, the integrity of mitochondrial function serves as the primary barrier to maintaining cellular homeostasis and preventing aberrant activation of the cGAS-STING pathway. In turn, aging itself promotes cumulative mitochondrial damage, which amplifies mtDNA leakage and cGAS-STING activation, forming a vicious cycle that accelerates both aging and diabetic complications (Wei et al., 2026). Given this central role, therapeutic strategies aimed at preserving mitochondrial integrity may hold the key to breaking this cycle and mitigating inflammation-driven metabolic diseases.

#### 2.1.1 ROS and the MPTP

Under hyperglycemic and lipotoxic conditions, mitochondrial electron transport chain (ETC.) activity becomes dysregulated, leading to a marked increase in ROS production (Ma et al., 2023a; Yan et al., 2022). Excessive ROS not only directly oxidize mtDNA—thereby rendering it more prone to escape from damaged mitochondria—but also promote the opening of the mitochondrial permeability transition pore (mPTP), which permits small molecules such as mtDNA to be released from the matrix into the cytosol (Li et al., 2023). Consequently, these ROS-driven events synergistically facilitate mtDNA leakage, a key trigger for activating the cGAS-STING innate immune pathway. In support of this, studies have demonstrated that the mitochondria-targeted antioxidant Mito-TEMPO effectively reduces mtDNA release and suppresses cGAS-STING activation, thereby alleviating diabetes mellitus-related atrial fibrillation and cardiomyopathy (Meng et al., 2026). Thus, preserving mtDNA integrity through the scavenging of mitochondrial ROS emerges as a viable strategy to interrupt this pathogenic signaling cascade, with mitochondrial ROS production itself representing a critical upstream intervention node. Notably, given that strategies aimed at enhancing mitochondrial quality control have already shown therapeutic promise in skeletal muscle pathophysiology (Jiang et al., 2026; Gong et al., 2026), it is plausible that such approaches could be extrapolated to combat diabetic complications in multiple tissues. Collectively, these insights underscore the need for further investigation into tissue-specific ROS–mTDP–cGAS–STING axis modulators as novel therapeutics for metabolic disease.

#### 2.1.2 Mitochondrial dynamics and quality control

Mitochondrial morphology and function are finely regulated by the processes of fusion and fission. In the diabetic state, this balance is disrupted. Research indicates that under high glucose and high-fat conditions, the expression of mitofusin 2 (MFN2) is downregulated, while the activity of fission proteins (such as DRP1) is abnormally increased, leading to mitochondrial fragmentation (Xiong et al., 2023; Wang et al., 2024a). This aberrant fission not only reduces mitochondrial function but also increases the risk of mtDNA leakage by promoting the oligomerization of voltage-dependent anion channel 1 (VDAC1) (Wang et al., 2024a). Moreover, ceramide synthase 6 (CerS6)-derived ceramide directly binds to VDAC1 at the Glu59 residue, triggering mtDNA leakage and activating the cGAS-STING pathway specifically in podocytes in diabetic kidney disease (Zhu et al., 2025). Inhibiting DRP1 activity or downregulating its expression significantly reduces mtDNA release and cGAS-STING activation, and ameliorates high glucose-induced vascular endothelial cell senescence (Xiong et al., 2023; Wang et al., 2024a). Additionally, mitophagy is a crucial quality control mechanism for clearing damaged mitochondria. In the diabetic environment, mitophagy is often inhibited, leading to the accumulation of damaged mitochondria and further exacerbating mtDNA leakage (Wang et al., 2025a; Zhou et al., 2025). Zhou et al. demonstrated that spermidine alleviates diabetic periodontitis by reversing senescence of human periodontal ligament stem cells (PDLSCs) via mitophagy-dependent clearance of mitochondrial-derived dsDNA, thereby blocking STING-TBK1 phosphorylation (Zhou et al., 2025). Hence, restoring the balance of mitochondrial dynamics and enhancing mitophagy are considered key strategies to suppress the cGAS-STING pathway. Evidently, maintaining the dynamic equilibrium of the mitochondrial network is crucial for curbing the aberrant release of mtDNA.

#### 2.1.3 The role of mitochondrial transcription factor A (TFAM)

TFAM is a key regulatory protein for mtDNA replication and transcription, and its levels directly impact mtDNA stability and packaging. Studies have found that TFAM expression is reduced in DKD (Chung et al., 2019) and diabetic retinopathy (Wang et al., 2025b). This reduction in TFAM leads to abnormal mtDNA packaging, rendering it more susceptible to oxidative damage and release from the mitochondria into the cytosol, thereby activating the cGAS-STING pathway (Chung et al., 2019). Restoring TFAM expression or function is considered an effective strategy to inhibit this pathway and reduce inflammation (Yun et al., 2026). For instance, the nuclear receptor FXR can improve mitochondrial function by upregulating TFAM, thereby suppressing the activation of the cGAS-STING pathway (Wang et al., 2025b). These findings establish TFAM as a key molecule for maintaining mtDNA-mitochondrial homeostasis and preventing autoinflammation. Thus, TFAM not only acts as a guardian of mitochondrial function but also serves as a crucial molecular switch linking mitochondrial homeostasis with the innate immune response.

To systematically illustrate the complexity of mitochondrial nucleic acid release, we summarize the major pathways of mtDNA leakage, their upstream triggers, relevant cell types, and key evidence in Table 1.

**TABLE 1:** Pathways of mitochondrial nucleic acid release in diabetic metabolic stress.

Release pathway	Upstream diabetic triggers	Relevant cell types	Key evidence
mPTP opening	Hyperglycemia, lipotoxicity, ROS overload	Cardiomyocytes, endothelial cells	The mPTP opening allows mtDNA release from matrix to cytosol (Li et al., 2023); Mito-TEMPO reduces mtDNA leakage and inhibits cGAS-STING in diabetic cardiomyopathy and atrial fibrillation (Meng et al., 2026)
VDAC1 oligomerization	DRP1-mediated mitochondrial fission, ROS	Endothelial cells, podocytes	DRP1 promotes VDAC1 oligomerization, facilitating mtDNA escape (Xiong et al., 2023; Wang et al., 2024a); CerS6-derived ceramide binds VDAC1 at Glu59 to initiate mtDNA leakage in podocytes (Zhu et al., 2025)
Mitophagy impairment	High glucose, oxidative stress	Periodontal ligament stem cells	Impaired mitophagy leads to accumulation of damaged mitochondria and mtDNA leakage (Wang et al., 2025a; Zhou et al., 2025); spermidine restores mitophagy to clear mtDNA and inhibit STING (Zhou et al., 2025)
TFAM downregulation	Hyperglycemia, metabolic stress	Kidney (DKD), retina (DR)	TFAM downregulation promotes oxidative mtDNA damage and cytosolic release, activating cGAS-STING (Chung et al., 2019), whereas FXR upregulates TFAM via ATF4/NRF1 to restore mitochondrial homeostasis and suppress this pathway (Wang et al., 2025b)

### 2.2 Nuclear DNA damage and micronucleus formation

In addition to mitochondrial sources, nuclear-derived DNA can also activate the cGAS-STING pathway. Hyperglycaemia-induced oxidative stress leads to nuclear DNA damage and the formation of micronuclei. Micronuclei are extra-nuclear bodies containing chromosomal fragments or whole chromosomes that are not incorporated into daughter nuclei during cell division; their fragile nuclear envelope is prone to rupture, thereby exposing genomic DNA fragments to the cytoplasm (Kirsch-Volders et al., 2020). In pancreatic β-cells in T1DM, overexpression of PLAGL1 induces oxidative stress and DNA damage, resulting in nuclear DNA leakage and micronucleus formation, which in turn activates the cGAS-STING pathway and promotes β-cell apoptosis and dysfunction (Li et al., 2026a; Li et al., 2024a). Furthermore, in DKD, deficiencies in DNA repair genes, such as *TET2*, lead to the accumulation of micronuclei and sustained activation of the cGAS-STING pathway (Liang et al., 2024). Consequently, maintaining genomic stability and nuclear envelope integrity is essential for preventing sterile inflammation triggered by nuclear DNA (Kirsch-Volders et al., 2020; Gauthier and Comaills, 2021). Collectively, these findings indicate that cytosolic leakage of DNA, whether of mitochondrial or nuclear origin, represents a critical event that triggers the inflammatory cascade mediated by the cGAS-STING pathway.

### 2.3 Endoplasmic reticulum stress and mitochondria-endoplasmic reticulum contacts (MERCs)

Endoplasmic reticulum (ER) stress is a common feature in diabetes mellitus. Physical contact sites between the ER and mitochondria, known as MERCs, play a crucial role in calcium (Ca^2+^) signalling and lipid metabolism (Barrera et al., 2021; Ji et al., 2025). In diabetic retinopathy, high glucose conditions promote excessive Ca^2+^ transfer from the ER to mitochondria via the activation of the IP3R1-GRP75-VDAC1 axis (Li et al., 2023). Subsequent mitochondrial calcium overload triggers the opening of the mPTP and the release of mtDNA, thereby activating the cGAS-STING pathway. Thus, disruptions in ER stress and MERCs function represent a critical hub linking metabolic stress to mitochondrial inflammation (Chen et al., 2026a). Targeting key molecules involved in MERCs, such as GRP75, or intervening in calcium transport, may represent promising novel strategies to inhibit the cGAS-STING pathway (Li et al., 2023; Zhao et al., 2022a). In summary, as the structural basis for ER-mitochondria communication, dysfunction of MERCs provides an additional regulatory dimension for cGAS-STING pathway activation under metabolic stress.

## 3 Role of the cGAS-STING pathway in diabetic complications

**FIGURE 2:**
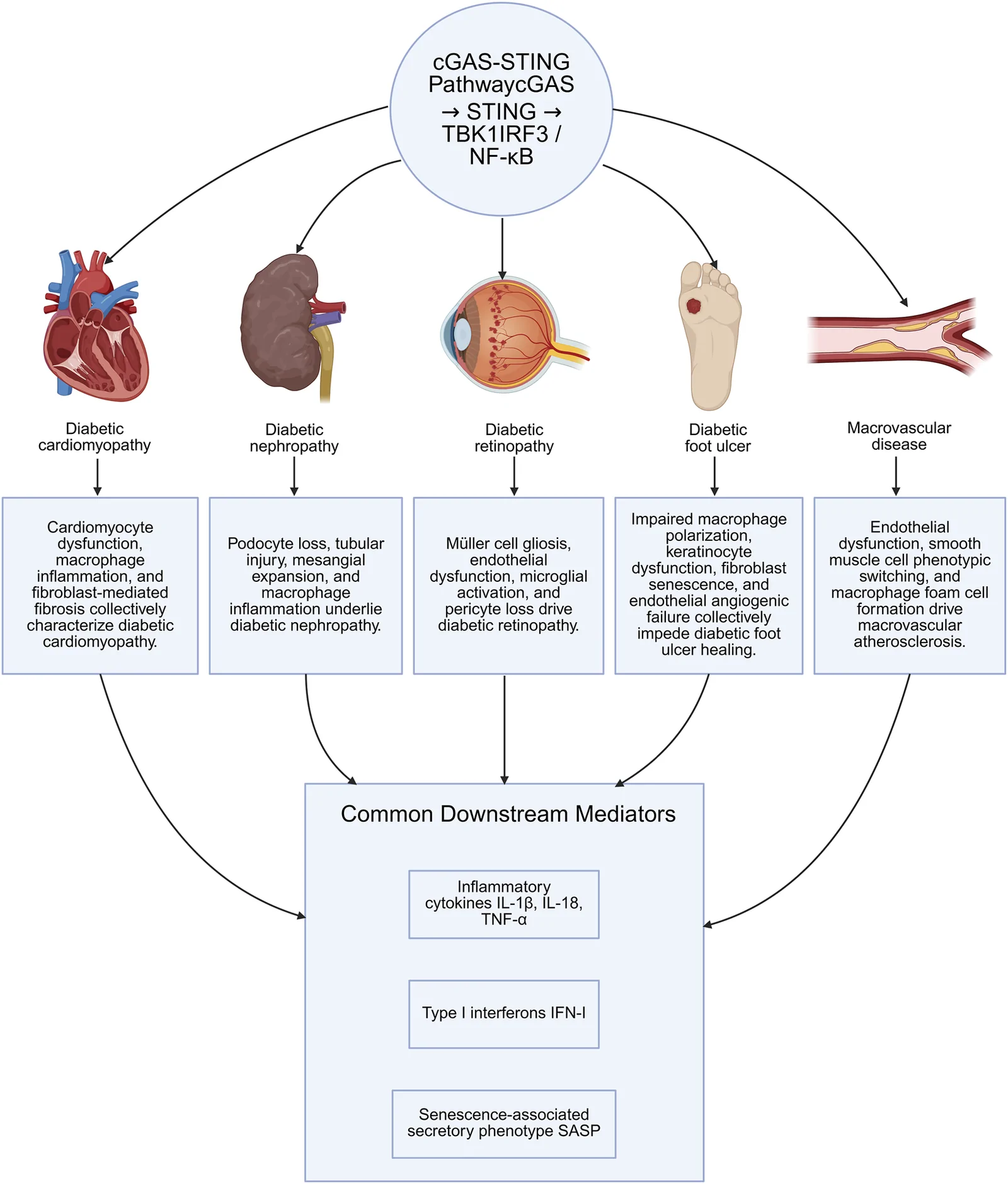
Schematic overview of the cGAS-STING signaling pathway and its pathogenic roles in diabetes mellitus and its major complications. The cGAS-STING cascade (cGAS → STING → TBK1 → IRF3/NF-κB) serves as a central hub that transduces signals from cytosolic DNA stressors—including mitochondrial DNA (mtDNA) leakage, metabolic stress, and autoimmune triggers—into downstream inflammatory and senescence responses. Activation of this pathway culminates in the production of common downstream mediators, including pro-inflammatory cytokines (IL-1β, IL-18, TNF-α), type I interferons (IFN-I), and senescence-associated secretory phenotype (SASP) factors. These effectors in turn drive tissue-specific pathological changes across diabetic complications: in diabetic cardiomyopathy, cardiomyocyte dysfunction, macrophage inflammation, and fibroblast-mediated fibrosis; in diabetic nephropathy, podocyte loss, tubular injury, mesangial expansion, and macrophage infiltration; in diabetic retinopathy, Müller cell gliosis, endothelial dysfunction, microglial activation, and pericyte degeneration; in diabetic foot ulcers, impaired macrophage polarization, keratinocyte dysfunction, fibroblast senescence, and endothelial angiogenic failure; and in macrovascular atherosclerosis, endothelial dysfunction, smooth muscle cell phenotypic switching, and macrophage foam cell formation. Collectively, this figure illustrates the cGAS-STING pathway as a unifying molecular driver linking sterile inflammation to the pathogenesis of diabetes and its multi-organ complications. Created in BioRender. (2026) https://BioRender.com/x66e0sw.

Aberrant activation of the cGAS-STING pathway has been widely documented in major complications of diabetes mellitus, where it plays a critical role in tissue inflammation, cell death, fibrosis, and functional impairment (Figure 2). Therefore, a thorough understanding of the regulatory mechanisms governing this pathway in various target organs is of great significance for elucidating the common pathological basis of diabetic complications.

### 3.1 DCM

DCM is a disorder of cardiac structure and function that occurs independently of coronary artery disease and hypertension, and represents a leading cause of heart failure in patients with diabetes mellitus. In recent years, numerous studies have demonstrated marked activation of the cGAS-STING signaling pathway in the cardiac tissue of DCM patients and animal models, where it directly participates in the pathological process of myocardial injury, thereby serving as a key nexus linking metabolic disturbances with cardiac inflammatory responses.

#### 3.1.1 Cardiomyocyte-intrinsic cGAS-STING activation

At the mechanistic level, hyperglycemic and lipotoxic environments can trigger excessive production of mitochondrial reactive oxygen species (mtROS) in cardiomyocytes, which subsequently causes oxidative damage to mtDNA and promotes its leakage into the cytoplasm. Once in the cytosol, this mtDNA acts as a DAMP recognized by cGAS, which in turn activates STING and its downstream IRF3 and NF-κB signaling axes, ultimately driving the expression of pro-inflammatory cytokines such as IL-1β and IL-18 (Ma et al., 2023a). Furthermore, activated STING signaling can also induce cardiomyocyte pyroptosis and pathological hypertrophy via an NLRP3 inflammasome-dependent mechanism, thereby exacerbating the deterioration of cardiac function (Yan et al., 2022). Importantly, the cGAS-STING pathway does not operate in isolation; rather, it intersects with a broader network of regulated cell death modalities—including apoptosis, necroptosis, ferroptosis, and cuproptosis—that collectively determine cardiomyocyte fate in ischemic and diabetic hearts (Qi et al., 2026a; Qi et al., 2026b). Recent evidence has further expanded our understanding of cardiomyocyte-intrinsic STING regulation. For instance, Sun et al. identified La-related protein 7 (LARP7) as a novel regulator of STING stability in cardiomyocytes (Sun et al., 2024). Under high-glucose conditions, LARP7 translocates from the nucleus to the cytoplasm, where it interacts with accumulated STING and inhibits its degradation via the autophagy-lysosomal pathway, thereby sustaining STING signaling and promoting myocardial fibrosis and apoptosis (Sun et al., 2024). Conversely, cardiomyocyte-derived USP20 has been shown to directly bind STING and promote its degradation through the autophagy pathway by deubiquitinating p62, consequently alleviating myocardial inflammation and improving ventricular remodeling (Zhou et al., 2026). Collectively, these findings have deepened our understanding of DCM pathogenesis from the perspectives of inflammation and cell death, and they strongly suggest that the cGAS-STING pathway may serve as a central hub that integrates diverse pathogenic signals.

Regarding regulatory factors, several molecules have been identified that influence the onset and progression of DCM through fine-tuning the cGAS-STING pathway. For instance, Metrnl is a hormone-like protein expressed in skeletal muscle and cardiac tissue, and its expression is significantly downregulated in the cardiac tissue of DCM models. Cardiac-specific overexpression of Metrnl enhances autophagy via activation of the LKB1/AMPK/ULK1 signaling pathway, promoting the degradation of STING protein, thereby inhibiting cGAS-STING pathway activation and significantly improving cardiac structure and function (Lu et al., 2023). Furthermore, BRG1 (Brahma-related gene 1), a key molecule involved in DNA double-strand break repair, is downregulated in the myocardium of DCM; its deficiency leads to abnormal accumulation of double-stranded DNA (dsDNA) in the cytoplasm, which in turn hyperactivates the cGAS-STING pathway, triggering cardiomyocyte inflammation and apoptosis (Chen et al., 2025a). Leptin, on the other hand, ameliorates high glucose-induced myocardial pathological changes by regulating Opa1-mediated mitochondrial fusion, improving mitochondrial oxidative damage, and suppressing aberrant activation of cGAS-STING signaling (Zhang et al., 2025a). Collectively, these studies reveal the potential value of modulating the cGAS-STING pathway in the prevention and treatment of DCM from multiple angles, providing a theoretical basis for identifying novel therapeutic targets.

#### 3.1.2 Macrophage-mediated inflammatory amplification

Beyond cardiomyocyte-intrinsic effects, macrophages play a critical role in amplifying cGAS-STING-driven cardiac inflammation in DCM. In the diabetic heart, cardiomyocyte-derived mtDNA can be released into the extracellular space and subsequently engulfed by resident cardiac macrophages, promoting their polarization toward the pro-inflammatory M1 phenotype and further amplifying inflammatory signaling (Meng et al., 2026). This cardiomyocyte-macrophage crosstalk creates a positive feedback loop: mtDNA-induced STING activation in cardiomyocytes triggers inflammatory cytokine production, which in turn activates macrophages, and these activated macrophages secrete additional pro-inflammatory mediators that exacerbate cardiomyocyte injury. Notably, macrophage-specific STING activation has been shown to promote M1 polarization and sustain local inflammation, while STING inhibition in macrophages can reverse this phenotype and attenuate cardiac inflammation (Yao et al., 2025). Thus, macrophage-mediated inflammatory amplification represents an important of cGAS-STING-driven pathology in DCM.

#### 3.1.3 Fibroblast activation and cardiac fibrosis

Cardiac fibrosis is a hallmark of DCM, and emerging evidence implicates the cGAS-STING pathway in fibroblast activation and extracellular matrix deposition. Fibroblasts do not directly sense metabolic stress as cardiomyocytes do; rather, they are activated by paracrine signals from injured cardiomyocytes. Notably, IL-37 has been shown to ameliorate myocardial fibrosis in DCM by preventing mitochondrial dysfunction and the release of mtDNA-containing vesicles from cardiomyocytes (Huang et al., 2024). These mtDNA-enriched vesicles are subsequently engulfed by cardiac fibroblasts, where they activate both TLR9 signaling and the cGAS-STING pathway, ultimately initiating a pro-fibrotic program and adverse cardiac remodeling (Huang et al., 2024). Once activated, fibroblasts upregulate the expression of fibronectin, α-SMA, and collagen type I/III, contributing to increased myocardial stiffness and diastolic dysfunction (Ma et al., 2023b). Moreover, inhibition of STING signaling in fibroblasts has been shown to reduce collagen deposition and mitigate cardiac fibrosis, highlighting the cGAS-STING pathway as a promising anti-fibrotic target in DCM (Hu et al., 2022).

#### 3.1.4 Endothelial contribution to diabetic cardiac dysfunction

The coronary microvascular endothelium is another key player in DCM pathogenesis, and cGAS-STING activation in endothelial cells contributes to cardiac dysfunction through multiple mechanisms. Hyperglycemia-induced mitochondrial dysfunction in endothelial cells leads to mtDNA leakage and cGAS-STING activation, which promotes endothelial apoptosis, migration dysfunction, and impaired angiogenesis (An et al., 2023). STING inhibition by C-176 has been shown to reverse high glucose-induced migration dysfunction and apoptosis in rat aortic endothelial cells, suggesting that endothelial STING activation directly contributes to microvascular rarefaction in the diabetic heart (An et al., 2023). Furthermore, endothelial STING signaling promotes the expression of adhesion molecules and pro-inflammatory cytokines, facilitating monocyte infiltration and perpetuating local inflammation (Mao et al., 2017). This endothelial dysfunction, combined with reduced capillary density, impairs myocardial oxygen supply and exacerbates the mismatch between supply and demand, ultimately contributing to diastolic dysfunction and heart failure progression in DCM patients.

In terms of interventional studies, administration of C-176, a specific STING inhibitor, effectively blocks cardiomyocyte apoptosis and inflammatory responses, offering a new pharmacological strategy for DCM treatment (Ma et al., 2023a). In the obesity-related DCM model established by Ma et al., C-176 was administered after the establishment of diabetes and cardiac dysfunction, and was shown to attenuate pre-existing inflammation and apoptosis (Ma et al., 2023a). Similarly, in the BRG1 knockdown DCM model, C-176 treatment partially reversed established cardiomyocyte inflammation and apoptosis *in vitro* (Chen et al., 2025a). Notably, exercise training, particularly high-intensity interval training (HIIT), has also been shown to mitigate myocardial inflammation, PANoptosis, and fibrosis in DCM model mice by suppressing cGAS-STING pathway activation, highlighting the potential of non-pharmacological interventions in modulating this pathway (Ma et al., 2025). Together, these findings suggest that targeting the cGAS-STING pathway holds promise as a novel breakthrough in the treatment of DCM, and that combined pharmacological and non-pharmacological intervention strategies may yield synergistic clinical benefits.

### 3.2 Diabetic kidney disease

DKD is the leading cause of end-stage renal disease (ESRD). Its pathogenesis is complex, involving damage to and interactions among various resident renal cells. Among these, glomerular podocytes and tubular epithelial cells (TECs) are the primary targets of DKD pathology. In recent years, a substantial body of research has demonstrated that the cGAS-STING pathway, a cornerstone of innate immunity, is aberrantly activated in these cells and plays a critical role in DKD progression. Consequently, this pathway has emerged as a highly promising therapeutic target.

#### 3.2.1 Podocyte injury

Significant activation of the cGAS-STING pathway in podocytes has been observed in renal biopsy tissues from DKD patients and across various DKD animal models. Mechanistic studies indicate that under conditions of obesity and diabetes mellitus, elevated circulating free fatty acids (lipotoxicity) induce mitochondrial damage in podocytes. This injury not only prompts the opening of the mPTP but also facilitates the leakage of mtDNA into the cytosol via BAX/BAK channels (Zang et al., 2022). Subsequently, this cytosolic mtDNA acts as a DAMP, directly binding and activating cGAS, which in turn activates the downstream STING protein. Notably, in DKD podocytes, STING activation primarily promotes inflammatory responses and podocyte injury through the TANK-binding kinase 1 (TBK1) and nuclear factor κB (NF-κB) signaling axis, rather than through interferon regulatory factor 3 (IRF3) (Zang et al., 2022). Indeed, STING activation alone is sufficient to directly induce podocyte loss and proteinuria (Mitrofanova et al., 2022). Correspondingly, genetic knockout of STING or pharmacological inhibition using agents like C-176 has been shown to significantly alleviate podocyte injury, proteinuria, and the decline in renal function in DKD model mice, further confirming the central pathogenic role of this pathway in DKD progression (Mitrofanova et al., 2022; Khedr et al., 2024). Therefore, targeting the STING signaling pathway holds promise as an effective strategy to halt podocyte injury and delay DKD progression.

#### 3.2.2 Tubular epithelial cell injury

Tubulointerstitial injury is a critical step in the progression of DKD to renal failure, with TECs being the core cells involved in this process. In DKD, TECs are also exposed to metabolic stressors such as hyperglycemia and lipotoxicity. Research has revealed that elevated expression of PCSK9 in DKD induces mtDNA damage and activates the cGAS-STING pathway in TECs, thereby exacerbating renal inflammation (Feng et al., 2023). This suggests that PCSK9 may serve as a crucial bridge connecting metabolic disturbances with the activation of renal innate immunity. Furthermore, recent studies have uncovered a novel mechanism involving interferon-stimulated gene 15 (ISG15) in TEC injury. Under DKD conditions, ISG15 expression is significantly upregulated and is closely associated with pyroptosis. Mechanistically, ISG15 forms a bidirectional positive feedback loop with STING: on one hand, STING signaling promotes ISG15 expression; on the other hand, ISG15 regulates STING stability through ISGylation. Together, they promote the activation of the mtDNA-STING-NLRP3 inflammasome axis, ultimately leading to TEC pyroptosis, inflammation, and fibrosis (Huang et al., 2025a). This discovery provides a novel perspective on the mechanisms determining TEC fate in DKD and suggests that disrupting the positive feedback loop between ISG15 and STING may hold therapeutic potential.

#### 3.2.3 Renal fibrosis

Renal fibrosis represents the common pathological feature of end-stage DKD, and mitochondrial dysfunction, along with sustained activation of the cGAS-STING pathway, are considered critical upstream events driving fibrosis. TFAM is a core protein responsible for maintaining mtDNA stability and mitochondrial function. Specific knockout of TFAM in TECs leads to severe mitochondrial dysfunction and persistent mtDNA leakage into the cytosol, which activates the cGAS-STING pathway, recruits immune cells, and releases substantial pro-inflammatory factors, ultimately resulting in severe renal fibrosis (Chung et al., 2019). Consequently, the TFAM-cGAS-STING axis is recognized as a key bridge linking mitochondrial dysfunction to renal inflammation and fibrosis, representing a potential core target for DKD therapy (Yun et al., 2026). Intervening in this axis—for instance, by enhancing mitophagy to clear damaged mitochondria or stabilizing TFAM—holds promise for suppressing the inflammatory cascade at its source, thereby effectively delaying the fibrotic process.

#### 3.2.4 Epigenetic regulation

In addition to the classical injury mechanisms described above, epigenetic regulation plays a significant role in the aberrant activation of the cGAS-STING pathway. Recent research has found that levels of RNA N6-methyladenosine (m6A) modification are significantly increased in the kidneys of DKD patients and in animal models. Notably, this hypermethylation is enriched on the mRNAs of *cGAS* and *STING1*, enhancing the stability of these transcripts. This leads to the overexpression of cGAS and STING proteins, thereby amplifying the activity of the entire cGAS-STING signaling pathway and ultimately exacerbating renal inflammatory and fibrotic responses (Tsai et al., 2024). This discovery not only reveals a novel epigenetic regulatory layer in the pathogenesis of DKD but also provides a solid theoretical foundation for developing novel DKD therapeutic strategies targeting RNA modifications.

### 3.3 Diabetic retinopathy (DR)

DR is a leading cause of blindness among working-age adults, characterized by a complex pathology that fundamentally involves dysfunction of the neurovascular unit, microvascular leakage, pathological neovascularization, and chronic low-grade inflammation. In recent years, the cGAS-STING signaling pathway has emerged as a critical bridge linking metabolic disturbance to sterile inflammation in DR pathogenesis, offering a pivotal direction for the exploration of novel therapeutic targets.

#### 3.3.1 Neuroinflammation and vascular leakage

In the early pathological progression of DR, Müller cells, the principal glial cells of the retina, serve as a key hub for inflammatory regulation. Specifically, the hyperglycaemic environment can induce mitochondrial dysfunction, leading to the leakage of mtDNA into the cytosol. This, in turn, activates the cGAS-STING pathway in Müller cells, triggering the release of inflammatory cytokines and exacerbating retinal neuronal and vascular damage (Li et al., 2023; Wang et al., 2025b). Furthermore, the specific activation of cGAS-STING signalling in retinal endothelial cells is a core driver of endothelial senescence, inflammatory responses, and capillary degeneration (Liu et al., 2023; Qin et al., 2026). Notably, genetic depletion or pharmacological inhibition of STING in diabetic animal models significantly alleviates these pathological changes, suggesting substantial potential for targeting this pathway to protect the retinal microcirculation. Further research indicates that the farnesoid X nuclear receptor (FXR) can upregulate the expression of TFAM by modulating the ATF4/NRF1 axis, thereby improving mitochondrial function and suppressing both mtDNA release and aberrant cGAS-STING pathway activation in Müller cells (Wang et al., 2025b). Consequently, concurrently targeting the cGAS-STING pathway in both Müller cells and endothelial cells may represent an effective strategy for intervening in the early pathological processes of DR.

#### 3.3.2 Cellular senescence

Endothelial cell senescence is recognized as a critical event in the onset and progression of early DR. Studies have demonstrated that hyperglycaemia-induced endothelial senescence is dependent on the activation of the mtDNA-cGAS-STING pathway, with an imbalance in mitochondrial dynamics playing a pivotal role. Mechanistically, inhibiting DRP1-mediated mitochondrial fission blocks mtDNA release, thereby suppressing the cGAS-STING pathway and delaying endothelial senescence (Wang et al., 2024a). This process involves the interaction of DRP1 with the mitochondrial channel protein VDAC1, which promotes VDAC1 oligomerization—a necessary step for mtDNA escape into the cytosol (Wang et al., 2024a). Subsequently, activation of the STING pathway drives cellular senescence and promotes the formation of the senescence-associated secretory phenotype (SASP) by inducing the phosphorylation of downstream effectors IRF3 and NF-κB. This amplifies local senescent signals into a global inflammatory milieu. These findings collectively highlight the central role of mitochondrial dynamics in maintaining endothelial homeostasis.

#### 3.3.3 Microglia and neurodegeneration

Microglia-mediated neuroinflammation is also critically implicated in the neurodegeneration associated with DR. The endogenous STING agonist cGAMP accumulates in the diabetic retina and can be transported into microglia via the P2RX7 transporter, where it activates STING signalling (Ge et al., 2025). Activated microglia subsequently disrupt the inner blood-retinal barrier (iBRB), leading to retinal neurodegeneration (Ge et al., 2025). Importantly, this discovery reveals that cGAMP functions not only as a signalling molecule but also as a “paracrine” messenger, transmitting and amplifying inflammatory signals between cells. Therefore, targeting P2RX7 or STING in microglia offers a novel therapeutic strategy to interrupt the vicious cycle of “inflammation-vascular leakage-neurodegeneration”. This approach holds promise as a potential intervention for DR, opening new avenues for protecting the retinal neurovascular unit in diabetes mellitus.

### 3.4 Diabetic foot ulcer (DFU)

DFU represents one of the most severe complications of diabetes mellitus, typically characterized by chronically non-healing wounds that frequently lead to limb amputation. Although multiple factors contribute to its pathogenesis, persistent inflammation and local immune dysregulation are considered core pathological mechanisms underlying the impaired healing in DFU. In recent years, the cGAS-STING signaling pathway has emerged as a critical bridge connecting metabolic stress with sterile inflammation, playing a pivotal regulatory role in the pathogenesis of DFU. Normal wound healing proceeds through distinct phases—hemostasis, inflammation, proliferation/granulation tissue formation, re-epithelialization, and remodeling. Therefore, a thorough understanding of the pathological mechanisms of this pathway in DFU is of great significance for the development of effective targeted therapeutic interventions.

#### 3.4.1 Hemostasis

The hemostatic phase is initiated immediately after injury, involving platelet aggregation, clot formation, and the release of growth factors and cytokines that orchestrate subsequent repair processes. In diabetic wounds, the hemostatic phase is often dysregulated due to platelet dysfunction and abnormal clotting (Bashir and Ali, 2018). Although direct evidence linking cGAS-STING activation to impaired hemostasis in DFU remains limited, emerging data suggest that STING activation in the epidermis of diabetic mice is associated with autophagy dysfunction, which delays wound healing from the earliest stages (Feng et al., 2022). Furthermore, the sustained activation of cGAS-STING in the diabetic wound microenvironment may contribute to aberrant platelet activation and thrombogenesis through the induction of type I interferons and pro-inflammatory cytokines, potentially disrupting the delicate balance between clot formation and fibrinolysis required for proper wound initiation.

#### 3.4.2 Inflammation

Under persistent stimulation by the metabolic stressor hyperglycemia, the cGAS-STING pathway becomes markedly activated in tissue-resident macrophages (Geng et al., 2023). This activation potently drives M1 (pro-inflammatory) macrophage polarization, which in turn triggers the release of large amounts of pro-inflammatory cytokines (e.g., IL-1β, TNF-α) and consequently establishes an inflammatory microenvironment detrimental to wound healing. Meanwhile, the same pathway simultaneously suppresses M2 (anti-inflammatory/repair) macrophage polarization, thereby blocking the anti-inflammatory responses and regenerative processes essential for tissue repair (Deng et al., 2024). Mechanistically, the massively generated ROS in the high-glucose environment activates STING signaling by inducing the escape of mtDNA to the cytoplasm, which further promotes the pro-inflammatory macrophage phenotype. In addition, PGE2—a downstream lipid mediator of inflammatory cascades—exerts context-dependent effects via its EP receptors and has also been implicated in the dysregulated wound microenvironment in diabetes (Yang et al., 2026). Notably, both STING and M1 macrophages are elevated in wound tissues from DFU patients and diabetic mice, and this elevation correlates with delayed wound closure. As a result, this immune polarization imbalance, mediated by the cGAS-STING pathway, represents a key driver of persistent wound inflammation and impaired healing.

#### 3.4.3 Proliferation and granulation tissue formation

Aberrant activation of the cGAS-STING pathway exerts direct detrimental effects on fibroblasts and endothelial cells, which are essential for wound repair. Studies have confirmed that activation of this pathway induces premature senescence in fibroblasts, severely impairing their critical capabilities for proliferation and migration, thereby hindering granulation tissue formation (Guan et al., 2026). In endothelial cells, high glucose-induced STING activation inhibits angiogenesis. Palmitic acid-induced mitochondrial damage and mtDNA release activate the cGAS-STING-IRF3 signaling pathway, which upregulates MST1 expression and subsequently suppresses YAP, thereby inhibiting endothelial proliferation, migration, and tube formation (Yuan et al., 2017). In summary, the cGAS-STING pathway acts as a core driver in the pathological progression of DFU by inducing dysfunction in multiple cell types critical for proliferation and granulation tissue formation.

#### 3.4.4 Re-epithelialization

In diabetic wounds, re-epithelialization is severely impaired, a process in which STING activation in the epidermis—exacerbated by autophagy disorder—plays a critical role in delaying healing (Feng et al., 2022). Notably, diabetic foot ulcers (DFUs) frequently arise in weight-bearing areas such as the plantar foot, where mechanical stress *per se* can activate the mechanosensitive ion channel Piezo1 on keratinocytes. Ma et al. have demonstrated that Piezo1 activation induces intracellular glucose overload and accumulation of advanced glycation end products (AGEs), which in turn trigger mitochondrial DNA (mtDNA) leakage into the cytosol and subsequently activate the cGAS–STING cascade. Indeed, Piezo1 is increasingly recognized as a key transducer that converts mechanical stimuli into inflammatory responses, and emerging evidence highlights its regulatory functions in tissue repair and regeneration (Xue et al., 2026). Furthermore, keratinocyte-specific deletion of Piezo1 reduces AGE deposition and preserves mitochondrial integrity, and ablation of STING yields comparable protective effects (Ma et al., 2026). Consequently, this dual “mechano-metabolic” insult not only exacerbates local wound inflammation but also perpetuates a vicious cycle that impedes healing.

#### 3.4.5 Remodeling

The final phase of wound healing involves the maturation and reorganization of the extracellular matrix, primarily mediated by myofibroblasts, to restore tissue strength and function. In diabetic wounds, remodeling is often impaired by excessive or disorganized collagen deposition. Although direct evidence in DFU is limited, studies across multiple fibrotic conditions have established that sustained STING signaling drives fibroblast-to-myofibroblast differentiation and pathological collagen accumulation via pathways involving PERK-eIF2α (Ge et al., 2024) and TGF-β1/Smad signaling (Gairola and Kaundal, 2025). Conversely, pharmacological inhibition of the cGAS-STING pathway promotes well-organized collagen deposition and improves vascular density in diabetic wound models, supporting the therapeutic potential of targeting this pathway to restore proper remodeling (Chen et al., 2025b). For instance, the CPP@Oct hydrogel, which suppresses the cGAS-STING pathway, accelerates wound closure, increases collagen deposition, and improves vascular density in animal models (Chen et al., 2025b). Furthermore, STING-deficient diabetic mice exhibit enhanced wound closure associated with increased collagen deposition (Audu et al., 2022). These findings suggest that while acute, transient STING activation may be required for proper remodeling, its sustained activation in diabetic wounds disrupts the balance of extracellular matrix turnover, contributing to impaired tissue architecture and function.

Given the central role of the cGAS-STING pathway in DFU, targeting this pathway has emerged as a promising therapeutic strategy. On one hand, cell-based therapies show significant potential. For instance, local treatment with STING-edited macrophages can effectively induce the conversion of wound macrophages from a pro-inflammatory M1 phenotype to an anti-inflammatory M2 phenotype, thereby ameliorating the inflammatory microenvironment, promoting angiogenesis and collagen deposition, and accelerating wound healing (Geng et al., 2023). On the other hand, various novel biomaterials are being developed to inhibit this pathway. For example, a gelatin methacryloyl (GelMA) hydrogel loaded with the aryl hydrocarbon receptor (AhR) agonist FICZ (GelMA-FICZ) significantly improves wound healing in diabetic mice by restoring mitochondrial function and inhibiting the activation of the cGAS-STING-NLRP3 inflammasome axis (Wang et al., 2025a). Wang et al. demonstrated that AhR deficiency suppresses mitophagy via impairment of the PINK1/Parkin pathway, leading to the accumulation of damaged mitochondria and subsequent mtDNA leakage into the cytosol. This mtDNA leakage then activates the cGAS-STING-NLRP3 inflammasome axis, promoting M1 macrophage polarization and sustained inflammation. Conversely, activation of AhR by FICZ restores mitophagy, clears damaged mitochondria, prevents mtDNA leakage, and thereby indirectly suppresses cGAS-STING pathway activation (Wang et al., 2025a). Additionally, an antimicrobial hydrogel modified with nanozymes (CeO_2_-Y@ZIF-8@Gel) has demonstrated excellent efficacy. It effectively inhibits cGAS-STING pathway activation by scavenging excessive reactive oxygen species and repairing damaged mitochondrial DNA, thereby regulating macrophage polarization toward the M2 phenotype and promoting angiogenesis, ultimately synergistically accelerating wound repair (Deng et al., 2024). Collectively, these studies indicate that precise modulation of the cGAS-STING pathway through diverse strategies to reshape the immune-vascular repair axis holds promise for providing breakthrough solutions for the treatment of DFU.

### 3.5 Macrovascular complications and other diabetic complications

The aberrant activation of the cGAS-STING pathway is not only involved in the pathogenesis of diabetic microvascular complications but has also garnered increasing attention for its role in macrovascular and a range of other diabetic complications. In macrovascular conditions such as diabetes-induced aortic endothelial dysfunction and atherosclerosis, the hyperglycaemic environment serves as a critical initiating factor. Studies have shown that hyperglycaemia can induce excessive production of mitochondrial ROS in endothelial cells, leading to mtDNA damage and its subsequent release into the cytoplasm. This cytosolic mtDNA, acting as a DAMP, effectively activates the cGAS-STING signalling pathway and its downstream effectors, IRF3 and NF-κB. This cascade ultimately results in impaired endothelial cell migration and increased apoptosis, thereby exacerbating vascular endothelial injury (An et al., 2023). Consequently, targeting the cGAS-STING pathway holds promise as a potential therapeutic strategy for managing diabetic macrovascular disease.

Furthermore, this pathway plays a significant role in the pathogenesis of diabetes mellitus-related atrial fibrillation. In models of diabetes mellitus-induced atrial fibrillation, cardiomyocytes under metabolic stress exhibit mitochondrial dysfunction and mtDNA leakage. This, in turn, activates the cGAS-STING pathway, promoting the polarisation of macrophages towards a pro-inflammatory M1 phenotype. These M1 macrophages release a large quantity of inflammatory cytokines, amplifying local inflammatory signals within the atria. This process ultimately leads to atrial electrical and structural remodelling, increasing susceptibility to atrial fibrillation (Meng et al., 2026). Thus, the cell-immune cell crosstalk mediated by the cGAS-STING pathway represents a crucial mechanism in the development of diabetic arrhythmias.

Notably, the pathogenic impact of the cGAS-STING pathway extends to various other diabetes mellitus-associated complications. For instance, in diabetic lung injury, activation of this pathway is closely linked to pyroptosis in pulmonary vascular endothelial cells (Gao et al., 2025). In diabetic periodontitis, the hyperglycaemic and inflammatory environment induces mitochondrial dysfunction and mtDNA leakage in human periodontal ligament stem cells, activating the cGAS-STING pathway and inducing stem cell senescence—a key factor contributing to the diminished tissue repair capacity in the periodontium (Zhou et al., 2025). Similarly, in diabetic dry eye disease, hyperglycaemia-induced mitochondrial damage and cytosolic mtDNA leakage in corneal epithelial cells activate the cGAS-STING pathway, driving ocular surface inflammation. Interventions with agents such as fenofibrate can suppress this pathway by improving mitochondrial function, thereby exerting therapeutic effects (Sun et al., 2025b). Additionally, emerging evidence suggests that the cGAS-STING pathway may be involved in the pathological processes of other diabetic complications, including diabetes mellitus-associated neurocognitive impairment (Preeti et al., 2024) and diabetic gastroparesis (Fan et al., 2026). These findings broaden the perspective on this pathway as a potential common therapeutic target across a spectrum of diabetic complications. In summary, the cGAS-STING pathway plays a central role as an immune-inflammatory hub in diabetes mellitus and its complications, and strategies targeting this pathway hold significant potential for clinical translation (Table 2).

**TABLE 2:** Evidence map of cGAS-STING activation in diabetes and complications.

Disease	Model system	Activation trigger	Key effector/phenotype	Evidence type
DCM	DCM mice, H9C2 cells	Lipotoxicity, mtROS, mtDNA leakage	Cardiomyocyte pyroptosis, hypertrophy, apoptosis	Direct
DCM	DCM mice, cardiomyocytes	LARP7-mediated STING stabilization	Myocardial fibrosis, apoptosis	Direct
DCM	DCM mice, cardiomyocytes	BRG1 deficiency, dsDNA accumulation	Cardiomyocyte inflammation, apoptosis	Direct
DCM	DCM mice, cardiomyocytes	Leptin downregulation, Opa1 dysfunction	Mitochondrial oxidative damage, cardiac remodeling	Indirect
DCM	DCM mice, macrophages	Cardiomyocyte-derived mtDNA engulfment	M1 macrophage polarization, inflammatory amplification	Direct
DCM	DCM mice, fibroblasts	mtDNA-enriched vesicles from cardiomyocytes	Fibroblast activation, collagen deposition, fibrosis	Direct
DKD	DKD patients, DKD mice, podocytes	Lipotoxicity, mtDNA leakage via BAX/BAK	Podocyte injury, proteinuria, NF-κB activation	Direct
DKD	DKD mice, podocytes	CerS6-derived ceramide binding VDAC1 (Glu59)	mtDNA leakage, podocyte injury	Direct
DKD	DKD mice, TECs	PCSK9 upregulation, mtDNA damage	Renal inflammation, cGAS-STING activation	Indirect
DKD	DKD mice, TECs	ISG15-STING positive feedback loop	TEC pyroptosis, fibrosis via NLRP3	Direct
DKD	DKD mice, TECs	TFAM knockout, mtDNA leakage	Severe renal fibrosis, immune cell recruitment	Direct
DKD	DKD patients, DKD mice	METTL3-mediated m6A methylation of cGAS/STING1 mRNA	Enhanced cGAS/STING expression, renal fibrosis	Indirect
DR	DR mice, Müller cells	Hyperglycemia, mtDNA leakage	Neuroinflammation, vascular leakage	Direct
DR	DR mice, endothelial cells	mtDNA-cGAS-STING activation	Endothelial senescence, capillary degeneration	Direct
DR	DR mice, microglia	cGAMP transport via P2RX7	iBRB breakdown, retinal neurodegeneration	Direct
DFU	DFU patients, diabetic mice, macrophages	Hyperglycemia, mtROS, mtDNA leakage	M1 macrophage polarization, impaired wound healing	Direct
DFU	Diabetic mice, keratinocytes	Piezo1 activation, glucose overload, AGEs	mtDNA leakage, cGAS-STING activation, delayed re-epithelialization	Indirect
DFU	Diabetic mice, fibroblasts	cGAS-STING activation	Fibroblast senescence, impaired granulation tissue	Indirect
DFU	Diabetic mice, endothelial cells	Palmitic acid, mitochondrial damage	Impaired angiogenesis via MST1-YAP inhibition	Direct
Macrovascular	Diabetic mice, aortic endothelial cells	Hyperglycemia, mtROS, mtDNA damage	Endothelial migration dysfunction, apoptosis	Direct
Atrial fibrillation	Diabetic mice, cardiomyocytes, macrophages	MQC dysregulation, mtDNA leakage	M1 macrophage polarization, atrial remodeling	Direct
Insulin resistance	Obese mice, human adipose tissue	Lipotoxicity, mtDNA leakage	Adipose tissue inflammation, insulin resistance	Indirect
β-cell dysfunction	T2DM mice, β-cells	High-fat diet, mtDNA leakage	β-cell senescence, SASP, dysfunction	Direct
T1DM β-cell apoptosis	T1DM NOD mice, β-cells	PLAGL1 overexpression, oxidative DNA damage	Nuclear DNA leakage, micronuclei, β-cell apoptosis	Direct

## 4 Association of the cGAS-STING pathway with core pathological processes in diabetes mellitus

In addition to its direct effects on target organs, the cGAS-STING pathway is deeply involved in two core pathological processes of diabetes mellitus: insulin resistance and pancreatic β-cell dysfunction.

### 4.1 Insulin resistance and adipose tissue inflammation

Obesity-associated adipose tissue inflammation is a key driver of insulin resistance, and the cGAS-STING pathway, as a critical hub of innate immune signaling, plays a central role in disrupting adipose tissue homeostasis. Notably, adipocytes themselves express both cGAS and STING, and in adipose tissues from obese mice and human subjects, this signaling pathway is markedly activated. Consequently, it further exacerbates inflammatory responses in both adipocytes and adipose-resident immune cells, thereby promoting the onset and progression of insulin resistance (Bai and Liu, 2021; Dumesic, 2026). Meanwhile, peroxisome proliferator-activated receptor α (PPARα) expression is downregulated in monocytes from diabetic patients; interestingly, treatment with the PPARα agonist fenofibrate alleviates monocyte inflammation by improving mitochondrial function and inhibiting mtDNA leakage, which in turn blocks aberrant activation of the cGAS-STING pathway (Dong et al., 2023). Beyond PPARα, the AMPK/SIRT1/PGC-1α signaling axis serves as another central hub that integrates energy sensing with mitochondrial biogenesis and inflammation, and its dysregulation has been firmly linked to insulin resistance and metabolic inflammation (Wang et al., 2026a; Chen et al., 2025c). Together, these findings not only reveal a novel mechanism by which PPARα regulates innate immunity but also provide a new theoretical basis for applying fenofibrate in the treatment of diabetic complications.

In diabetic models: RBM43, an RNA-binding protein induced by inflammatory cytokines in adipocytes, suppresses mitochondrial biogenesis by inhibiting PGC1α translation; adipocyte-selective Rbm43 disruption in obese mice improves glucose tolerance, reduces adipose inflammation, and suppresses cGAS-STING activation (Dumesic et al., 2025). Bai et al. demonstrated that fat-specific DsbA-L knockout impairs mitochondrial function and promotes mtDNA release, activating the cGAS-cGAMP-STING pathway and inflammatory responses, whereas fat-specific DsbA-L overexpression protects against HFD-induced pathway activation (Bai et al., 2017). Macrophage PTP1B suppresses the JAK2/STAT3–OPA1 axis, causing mitochondrial fragmentation and mtDNA leakage, which activates cGAS/STING signaling and promotes a pro-inflammatory phenotype (Lei et al., 2025). In monocytes, PPARα expression is downregulated in diabetic patients; PPARα knockout increases cytosolic mtDNA release and activates the cGAS-STING pathway in monocytes under diabetic conditions, and STING knockout or STING inhibitor attenuates this monocyte activation (Dong et al., 2023). Collectively, these findings underscore that diverse cell types converge on mtDNA-driven cGAS-STING activation as a common pathogenic axis in obesity and diabetes, highlighting this pathway as a promising therapeutic target for metabolic inflammation.

**FIGURE 3:**
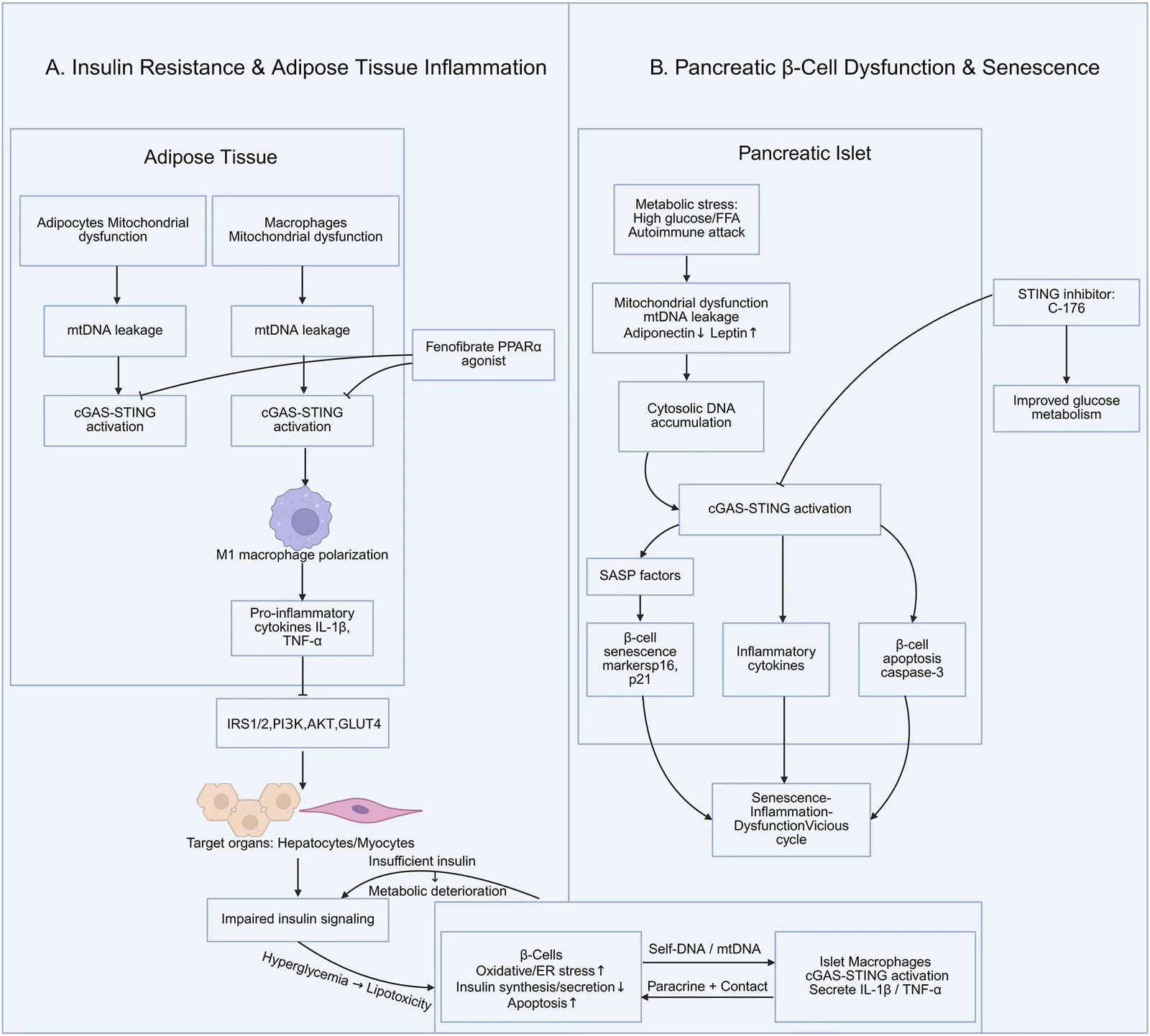
cGAS-STING-mediated mechanisms in insulin resistance, adipose tissue inflammation, and pancreatic β-cell dysfunction. **(A)** In adipose tissue, mitochondrial dysfunction in both adipocytes and adipose tissue macrophages leads to mtDNA leakage and subsequent cGAS-STING activation, which promotes M1 polarization of macrophages and secretion of IL-1β and TNF-α. These pro-inflammatory cytokines impair insulin signaling in target organs (hepatocytes and myocytes) by disrupting the IRS1/2–PI3K–AKT–GLUT4 axis, resulting in peripheral insulin resistance. Hyperglycemia and lipotoxicity further feed back to exacerbate adipose inflammation, establishing a vicious cycle. **(B)** In pancreatic islets, metabolic stress (high glucose, free fatty acids, and autoimmune attack) and mitochondrial dysfunction cause cytosolic DNA accumulation and cGAS-STING activation, which upregulates SASP factors and senescence markers (p16, p21) in β-cells, leading to oxidative/ER stress, reduced insulin synthesis and secretion, and increased apoptosis. Adipose tissue-derived signals—declining adiponectin and rising leptin—further aggravate islet pathology. Pharmacological STING inhibition (e.g., with C-176) attenuates these deleterious effects and improves glucose metabolism, highlighting the therapeutic potential of targeting this pathway. Together, these findings position cGAS-STING signaling as a critical node linking adipose inflammation, insulin resistance, and β-cell failure in the progression of type 2 diabetes. Created in BioRender. (2026) https://BioRender.com/lmn6bh4.

In steatohepatitis models, mtDNA-cGAS-STING pathway activation is directly linked to insulin resistance (HOMA-IR) and fibrosis, and combined targeting of this axis with a mitophagy enhancer and STING inhibitor yields superior multi-domain control of disease (Hamad et al., 2026). Research has demonstrated that liver-specific knockout of DRP1 (Dnm1l), a key protein involved in mitochondrial fission, leads to the formation of giant mitochondria and mitochondrial dysfunction, which in turn activates the cGAS-STING signaling pathway and exacerbates hepatic inflammation in alcoholic hepatitis (Ma et al., 2023c). Given that non-alcoholic fatty liver disease (NAFLD) is also characterized by mitochondrial dysfunction and chronic inflammation, this mechanism may similarly contribute to the pathogenesis and progression of NAFLD (Bai and Liu, 2021; Tian and Li, 2025). In summary, in the context of insulin resistance, whether through lipotoxicity-induced mitochondrial damage or mtDNA leakage resulting from imbalanced mitochondrial dynamics, activation of the cGAS-STING pathway plays a critical role in the inflammatory responses of adipose tissue and the liver. Targeting this pathway may therefore represent a potential therapeutic strategy for improving obesity-associated metabolic inflammation and insulin resistance (Figure 3).

### 4.2 Pancreatic β-cell dysfunction and senescence

The reduction in pancreatic β-cell mass and functional failure represent core pathological features in the progression of type 2 diabetes mellitus (T2DM). Recent studies have revealed that β-cells undergo accelerated senescence under metabolic stress, and this phenomenon has emerged as a critical driver of T2DM progression (Tsvetkov et al., 2022). Mechanistically, β-cell senescence induced by metabolic stressors—such as a high-fat diet—is fundamentally linked to mitochondrial dysfunction and the leakage of mitochondrial DNA (mtDNA). Once released into the cytoplasm, mtDNA acts as a damage-associated molecular pattern (DAMP), which is recognized by and activates the cGAS-STING signaling pathway (Tsvetkov et al., 2022). Activated STING not only exacerbates the local inflammatory microenvironment within islets by inducing the senescence-associated secretory phenotype (SASP), but also directly impairs β-cell function, thereby forming a vicious cycle of “senescence–inflammation–dysfunction”.

Cellular senescence and the SASP are now recognized as common pathological drivers across multiple organ systems, and emerging evidence indicates that impaired regeneration in aging and diabetes shares similar molecular underpinnings (Gong et al., 2026; Li et al., 2026b). Upon activation of the cGAS-STING pathway, downstream TBK1/IRF3 and NF-κB signaling cascades are initiated, which subsequently promote the expression of various inflammatory cytokines and intensify β-cell stress and dysfunction. Notably, intervention with STING inhibitors (e.g., C-176) effectively alleviates senescence-associated inflammatory responses and β-cell dysfunction, leading to significant improvement in glucose metabolism (Tsvetkov et al., 2022). This finding not only establishes the cGAS-STING pathway as a core driver in β-cell senescence, but also suggests STING as a potential therapeutic target for delaying T2DM progression.

Importantly, the cGAS-STING pathway also plays a critical role in the context of type 1 diabetes mellitus (T1DM). For instance, research has demonstrated that in T1DM, overexpression of pleiomorphic adenoma gene-like 1 (PLAGL1) induces oxidative stress and DNA damage, leading to the accumulation of cytosolic DNA fragments, which in turn activates the cGAS-STING pathway and ultimately promotes β-cell apoptosis (Li et al., 2026a). This mechanism reveals that, even in autoimmunity-driven T1DM, endogenous DNA damage similarly amplifies inflammatory responses through the cGAS-STING pathway and directly contributes to β-cell death. Therefore, whether in T2DM—primarily characterized by metabolic stress—or in T1DM—defined by autoimmunity—aberrant activation of the cGAS-STING pathway serves as a crucial hub that links cellular stress, inflammation, and β-cell fate determination.

## 5 Challenges

**FIGURE 4:**
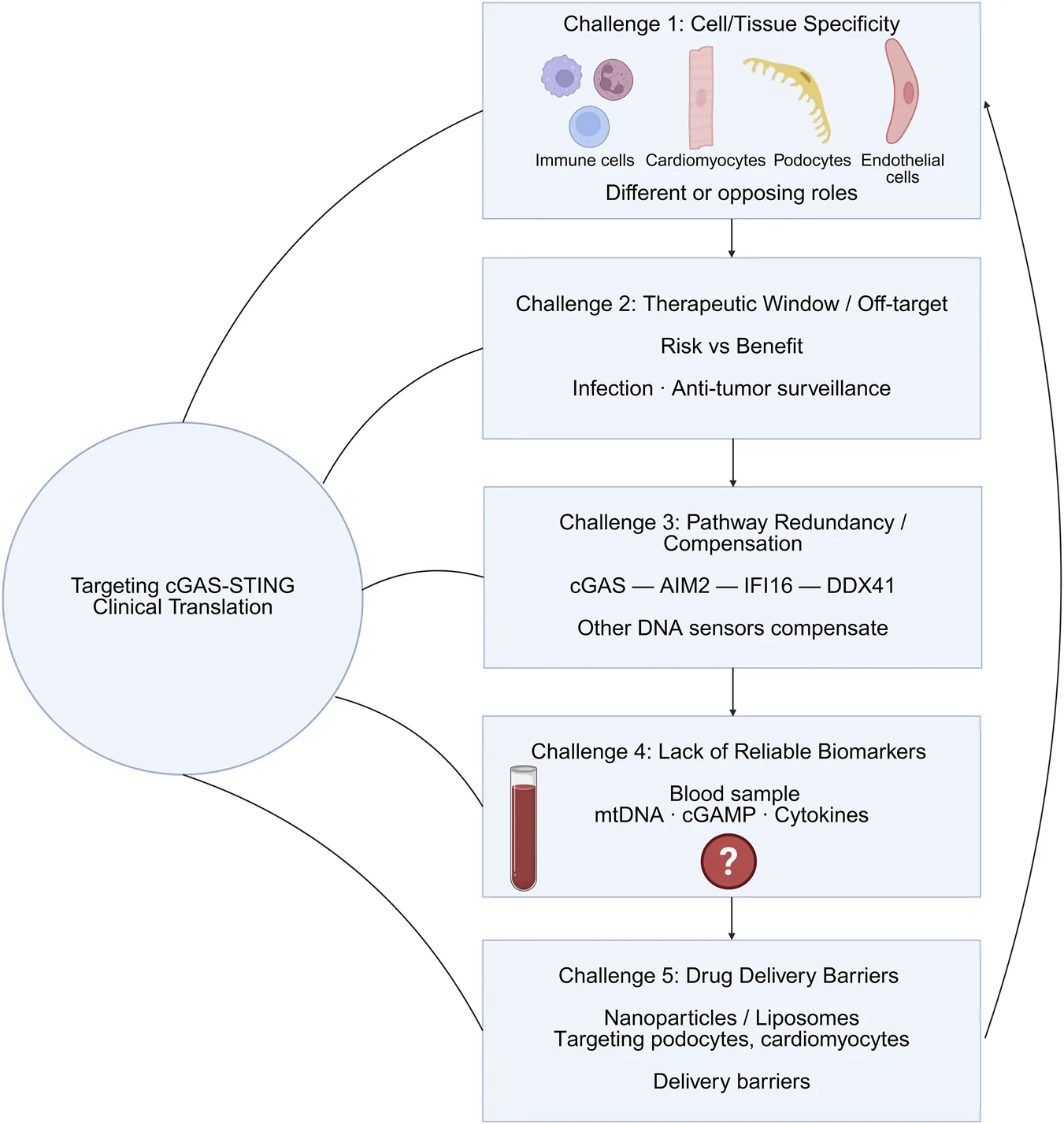
Key challenges for targeting the cGAS-STING pathway in diabetic complications. Five major hurdles are identified: Challenge 1 - Cell and tissue specificity (immune cells, cardiomyocytes, podocytes, and endothelial cells exhibit differential responses); Challenge 2 - Therapeutic window and off-target risks (potential impairment of infection surveillance and anti-tumor immunity); Challenge 3 - Pathway redundancy (other DNA sensors such as AIM2, IFI16, and DDX41 may compensate for cGAS-STING inhibition); Challenge 4 – Lack of reliable biomarkers (circulating mtDNA, cGAMP, and cytokines require validation in large-scale cohorts); Challenge 5 – Drug delivery barriers (nanoparticle- or liposome-based systems are needed to target cells such as podocytes and cardiomyocytes). Created in BioRender. (2026) https://BioRender.com/wuuh818.

Although the cGAS-STING pathway holds significant promise as a therapeutic target for diabetes mellitus, several challenges must be addressed before its clinical translation can be realized (Figure 4).

### 5.1 Cellular and tissue specificity

The role of the cGAS-STING pathway may differ, or even be opposing, across various stages of diabetes mellitus, as well as across different tissues and cell types. For instance, STING activation can be beneficial in the context of acute inflammation (e.g., antiviral responses) but detrimental during chronic inflammation (Kwak et al., 2025). Therefore, a more refined understanding of its spatiotemporal functions within specific cell types is essential for achieving precise intervention. Existing studies indicate that activation of this pathway in endothelial cells exacerbates diabetic macrovascular complications (An et al., 2023) and retinal neurovascular damage (Qin et al., 2026), whereas in cardiomyocytes, it mediates lipotoxicity-induced apoptosis and inflammation (Ma et al., 2023a). Furthermore, its role in immune cells is complex: while activation in macrophages can promote M1 polarization and exacerbate inflammation (Geng et al., 2023), moderate STING signaling may be beneficial for host defense under specific conditions. Consequently, in-depth analysis of the functional differences of the cGAS-STING pathway across diverse cell types—such as cardiomyocytes versus fibroblasts, or M1 versus M2 macrophages—is critical for developing precise intervention strategies. Only by accurately mapping its spatiotemporal dynamics at the single-cell level can we avoid the therapeutic blind spots associated with a “one-size-fits-all” intervention approach.

### 5.2 Therapeutic window and off-target effects

The cGAS-STING pathway serves as a crucial defense line against pathogenic infections. Systemic, long-term inhibition of this pathway could increase host susceptibility to viral and bacterial infections or compromise normal immune surveillance functions, such as anti-tumor immunity. Indeed, this pathway plays a central role in defending against DNA viruses (Zhang et al., 2022) and maintaining anti-tumor immunity (Vornholz et al., 2023). Therefore, identifying the optimal therapeutic window and administration strategy (e.g., local delivery) to balance efficacy and safety is an issue that must be addressed. Notably, some studies have begun exploring strategies to mitigate the risks associated with systemic inhibition through localized drug delivery (e.g., topical application of inhibitor-laden gels to wounds) or the use of specialized delivery systems (Deng et al., 2024), offering new avenues to overcome this challenge. Thus, future clinical translation will require a nuanced balance between precisely timed intervention and optimized drug delivery to maximize therapeutic benefit while minimizing safety risks.

### 5.3 Pathway redundancy and compensation

Several intracellular DNA sensors exist, including AIM2, IFI16, DDX41, ZBP1, and TLR9, and these receptors may exhibit functional redundancy and compensatory mechanisms. Consequently, inhibiting only cGAS or STING might not suffice to completely block inflammatory responses mediated by other DNA sensors (Kwak et al., 2025). In human keratinocytes, for instance, both cGAS and IFI16 are required for full activation of the innate immune response to exogenous DNA and DNA viruses; moreover, IFI16 is also essential for cGAMP-induced STING activation, as it interacts with STING to promote its phosphorylation and translocation (Almine et al., 2017). Similarly, in human macrophages, IFI16 operates on two distinct levels: its depletion impairs cGAMP production upon DNA stimulation, whereas its overexpression amplifies cGAS function; furthermore, IFI16 is critical for downstream signaling triggered by cGAMP, facilitating the recruitment and activation of TBK1 within the STING complex (Jonsson et al., 2017). This intricate interplay highlights that therapeutic strategies targeting only the cGAS–STING core may be undermined by the remaining sensor network, necessitating combinatorial approaches.

Beyond IFI16, the AIM2 inflammasome pathway also intersects with the cGAS–STING axis in disease contexts such as diabetic kidney disease (DKD). In diabetic nephropathy, AIM2 expression is markedly elevated in renal tubules from DN patients and db/db mice, and AIM2-mediated pyroptosis contributes to proximal tubular epithelial cell injury (Kwak et al., 2025; Li et al., 2024b). Likewise, DDX41 is required for cGAS–STING activation against DNA viruses, functioning upstream of cGAS by modulating dsDNA/ssDNA homeostasis through its unwinding and annealing activities (Singh et al., 2022). ZBP1, in turn, represents a downstream effector that integrates cGAS–STING signals with necroptotic pathways; indeed, STING upregulates both ZBP1 and MLKL, and STING-driven ZBP1-mediated necroptosis has been established as a central pathogenic mechanism in caspase-8-deficient inflammation and STING-associated vasculopathy with onset in infancy (SAVI) (Kelepouras et al., 2025). TLR9, an endosomal DNA sensor, further contributes to this redundancy. For example, in diabetic cardiomyopathy, mtDNA-rich vesicles released from cardiomyocytes are engulfed by fibroblasts, activating both TLR9 and cGAS–STING pathways to promote fibrosis (Huang et al., 2024). Collectively, these findings underscore a multilayered sensor web in which AIM2, DDX41, ZBP1, and TLR9 not only parallel but also reinforce cGAS–STING signaling, amplifying pathogenic outcomes across diverse tissue contexts.

Moreover, this functional redundancy is not confined to DNA sensors—it also extends to downstream cell death pathways, where apoptosis, necroptosis, pyroptosis, and ferroptosis form an interconnected network with extensive crosstalk, thereby further complicating single-target therapeutic strategies (Gong et al., 2026; Qi et al., 2026a). Consequently, future therapeutic approaches may need to consider targeting multiple key DNA sensors or their common downstream signaling nodes to achieve more comprehensive control of inflammation. Taken together, these observations suggest that moving beyond the paradigm of a single target toward multi-target interventions that converge on critical nodes within the signaling network may represent an effective strategy to navigate this complex regulatory landscape.

### 5.4 Lack of biomarkers

There is currently a scarcity of specific and sensitive biomarkers to monitor the activation status of the cGAS-STING pathway in diabetic patients, which hinders patient stratification and assessment of treatment responses. Circulating levels of mtDNA and expression levels of downstream effectors of STING are potential candidate biomarkers, but they require validation in large-scale clinical studies. Some research has begun exploring these possibilities; for instance, elevated plasma mtDNA levels (Kwak et al., 2025) and changes in the expression of STING pathway-related genes (such as ZBP1) in monocytes (Esawie et al., 2025) have been associated with disease status in patients with T2DM. However, translating these findings into clinically useful diagnostic tools requires overcoming challenges related to standardization of sample processing, harmonization of detection methods, and validation in large, prospective cohorts. Therefore, developing a well-validated, multi-omics-based biomarker panel that dynamically reflects pathway activity is a necessary bridge for translating precision targeting of this pathway from concept to clinical practice.

### 5.5 Drug delivery hurdles

Efficiently and specifically delivering small molecule inhibitors or nucleic acid-based therapeutics to target cells (e.g., podocytes, cardiomyocytes) represents a significant challenge. Nanomedicine delivery systems offer a potential solution, yet their *in vivo* stability and biosafety require further optimization. Currently, various nanoplatforms are being developed for targeted delivery of STING inhibitors or agonists (Deng et al., 2024). Examples include the use of hydrogel-loaded nanozymes for immunomodulation in diabetic wounds (Deng et al., 2024), and the construction of responsive nanomaterials for precision delivery to specific tissues (He et al., 2023). These studies provide promising strategies to overcome drug delivery hurdles; however, further research is needed to enhance targeting efficiency, biocompatibility, and clinical translational feasibility. It is foreseeable that with continued breakthroughs in materials science and engineering, the development of next-generation delivery systems characterized by high targeting specificity, controlled release, and favorable safety profiles will be pivotal in unlocking the full therapeutic potential of this pathway.

## 6 Therapeutic strategies

**FIGURE 5:**
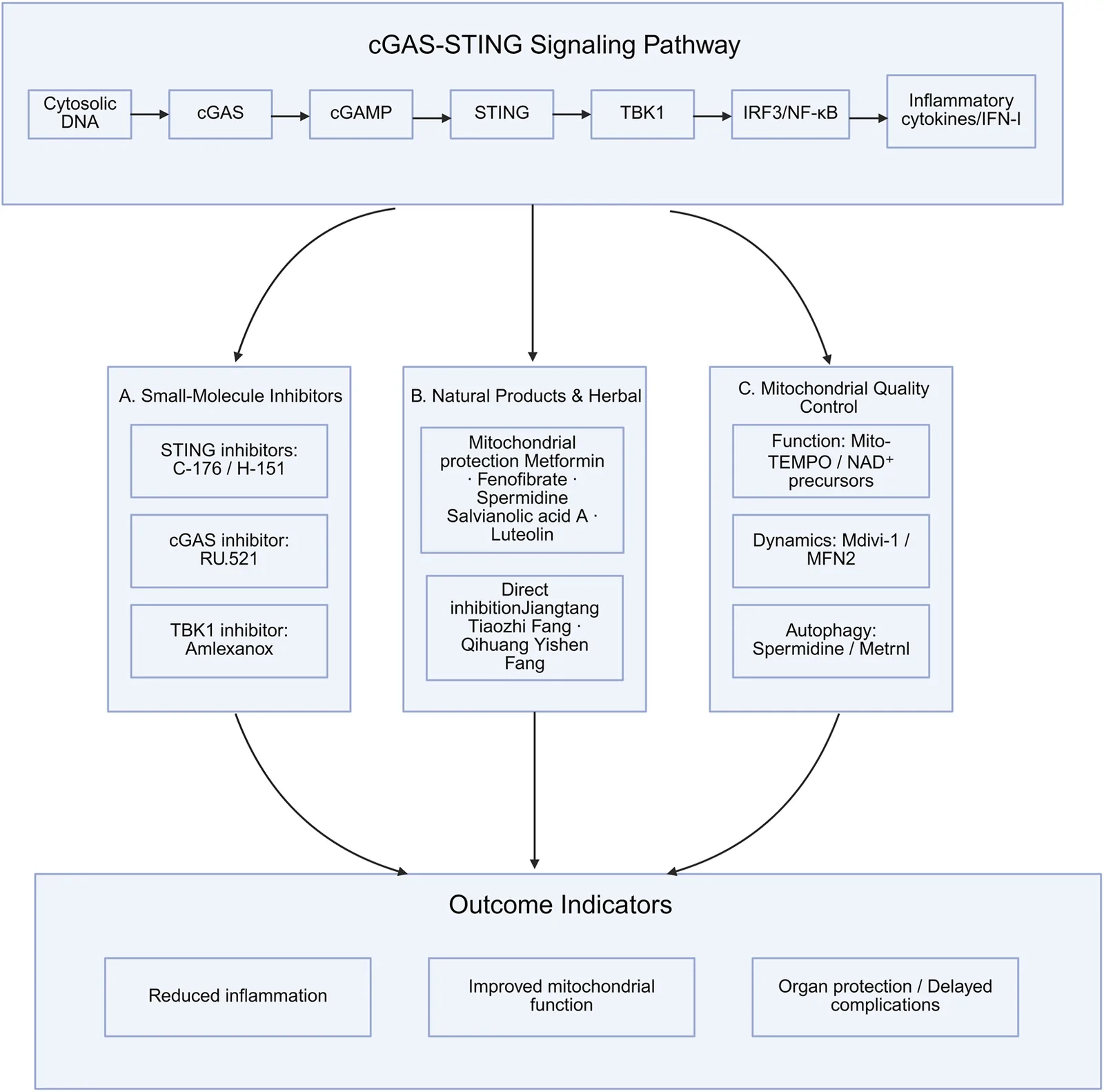
Therapeutic strategies targeting the cGAS-STING pathway in diabetic complications. Upon cytosolic DNA accumulation, cGAS synthesizes cGAMP, which activates STING, followed by TBK1 and downstream IRF3/NF-κB signaling, leading to the production of inflammatory cytokines and type I interferons. Therapeutic approaches include: **(A)** small-molecule inhibitors (STING inhibitors C-176 and H-151, cGAS inhibitor RU.521, and TBK1 inhibitor amlexanox); **(B)** natural products and herbal formulas (metformin, fenofibrate, spermidine, salvianolic acid A, luteolin, Jiangtang Tiaozhi Formula, and Qihuang Yishen Formula); and **(C)** mitochondrial quality control interventions (Mito-TEMPO, NAD^+^ precursors, the DRP1 inhibitor Mdivi-1, modulation of MFN2, spermidine, and Metrnl). Collectively, these strategies reduce inflammation, improve mitochondrial function, provide organ protection, and delay the progression of diabetic complications. Created in BioRender. (2026) https://BioRender.com/qw7kl7x.

In response to the aforementioned challenges, researchers are actively exploring various therapeutic strategies targeting the cGAS-STING pathway (Figure 5).

### 6.1 Small molecule inhibitors

Given the central role of the cGAS-STING pathway in the pathogenesis of diabetes mellitus and its complications, the development of inhibitors targeting key nodes within this pathway has emerged as a highly promising therapeutic strategy. To date, several small molecule inhibitors have demonstrated significant efficacy in preclinical models, effectively alleviating inflammatory responses and tissue damage by blocking either the initial activation or downstream signaling of the pathway (He et al., 2024a; Cai et al., 2025). Based on their targets, these inhibitors can be categorized into three main classes.

#### 6.1.1 STING inhibitors

STING, a core adaptor protein in the pathway, represents an ideal target for pharmacological intervention. Among these, C-176 and H-151 are the most extensively characterized classical STING palmitoylation inhibitors. They function by covalently binding to the transmembrane domain of STING, thereby preventing its palmitoylation and the formation of high-molecular-weight multimers, which effectively blocks STING activation and its downstream signaling cascades (Ma et al., 2023a; Feng et al., 2023). Both inhibitors have shown potent protective effects in various preclinical models of diabetic complications. For instance, in a DCM mice model, intervention with C-176 significantly inhibited cardiomyocyte apoptosis and inflammatory responses (Ma et al., 2023a). Similarly, in DKD mice models, C-176 effectively alleviated renal inflammation (Feng et al., 2023). In DR, treatment with a STING inhibitor or genetic knockout of STING effectively suppressed retinal endothelial cell senescence and inflammation, thereby preventing the progression of early retinopathy (Liu et al., 2023). Furthermore, in studies on DFU, pharmacological or genetic inhibition of STING promoted macrophage polarization towards the anti-inflammatory M2 phenotype, thereby accelerating wound healing (Wang et al., 2025a). Collectively, these findings establish STING as a highly promising key target for the treatment of diabetic complications.

#### 6.1.2 cGAS inhibitors

cGAS, functioning as the primary “sensor” of the pathway, acts upstream of STING. Directly inhibiting cGAS activity can block pathway activation at its source, making this a highly attractive strategy. RU.521 is a potent and selective cGAS inhibitor that directly binds to cGAS, competitively inhibiting its interaction with DNA or reducing its catalytic activity, thereby decreasing the production of the second messenger cGAMP. In a type 1 diabetes mellitus (T1DM) mice model, inhibition of cGAS with RU.521 effectively protected pancreatic beta cells, mitigated autoimmune attacks, and slowed disease progression (Li et al., 2026a). Additionally, studies have indicated that in DCM mice, the downregulation of the DNA damage repair protein BRG1 leads to the accumulation of cytosolic dsDNA, subsequently activating the cGAS-STING pathway. Treatment with RU.521 significantly ameliorated the inflammation and apoptosis in cardiomyocytes induced by BRG1 knockdown, further substantiating the therapeutic potential of targeting cGAS (Chen et al., 2025a). Therefore, targeting cGAS offers a sound therapeutic rationale for intervening in the inflammatory cascade of diabetic complications at its origin.

#### 6.1.3 TBK1 inhibitors

TBK1 is a critical kinase downstream of STING, responsible for phosphorylating and activating the transcription factor IRF3, which in turn induces the expression of type I interferons and pro-inflammatory cytokines. Consequently, targeting TBK1 can also effectively abrogate downstream signal transmission. Research has shown that the TBK1/IKKε inhibitor amlexanox improves cardiac function and reduces inflammation in cardiovascular disease models. In rats with acute myocardial infarction, amlexanox treatment increased left ventricular ejection fraction, reduced myocardial inflammatory response, and decreased cardiomyocyte apoptosis (Mo et al., 2023). In Western diet-fed Ldlr^−/−^ mice, amlexanox ameliorated dyslipidemia, inflammation, and vascular dysfunction, and prevented atherosclerosis development (Zhao et al., 2022b). Furthermore, amlexanox significantly alleviated pressure overload-induced cardiac hypertrophy and myocardial fibrosis by inhibiting TBK1 activity (Cai et al., 2025; Xiao et al., 2024). This finding suggests that, in addition to targeting cGAS and STING, targeting the downstream node TBK1 may offer a novel therapeutic avenue for diabetic cardiovascular complications. Moreover, given the established clinical experience with amlexanox, its translational potential is noteworthy. Thus, targeting TBK1 not only validates the feasibility of targeting downstream signaling nodes but also opens new avenues for drug repurposing.

In summary, pharmacological interventions targeting distinct nodes within the cGAS-STING pathway have demonstrated significant efficacy across various preclinical models of diabetic complications (Table 3). These research findings not only deepen our understanding of the pathogenesis underlying diabetic complications but also provide a robust theoretical foundation and potential drug candidates for the development of novel therapeutic strategies. Moving forward, with further elucidation of the mechanisms of action of these inhibitors and the advancement of their clinical translation, targeting the cGAS-STING pathway holds great promise for achieving significant breakthroughs in the treatment of diabetic complications.

**TABLE 3:** Summary of small molecule inhibitors targeting the cGAS-STING pathway in diabetic complications.

Compound	C-176	H-151	RU.521	Amlexanox
Target	STING	STING	cGAS	TBK1/IKKε
Mechanism	Covalent binding to mouse STING Cys91; prevents palmitoylation and multimerization	Covalent binding to STING transmembrane domain; prevents palmitoylation	Binds at cGAS/dsDNA complex interface; inhibits cGAS enzymatic activity	Inhibits TBK1/IKKε kinase activity; blocks IRF3 phosphorylation
Model & Disease	DCM mice DKD mice	T1DM NOD mice	T1DM NOD mice DCM mice (BRG1 KD)	AMI rats (i.p) Ldlr^−/−^ mice (atherosclerosis)
Main Outcome	Inhibited cardiomyocyte apoptosis; alleviated renal inflammation	Preserved β-cell mass; improved glucose homeostasis	Protected β-cells; ameliorated cardiomyocyte inflammation/apoptosis	Improved LVEF, reduced inflammation; ameliorated dyslipidemia, prevented atherosclerosis
Limitations	Mouse-specific; no activity on human STING	Inactive in human PBMCs; palmitoylation site not essential for human STING	Less potent on human cGAS	Kinase target; potential off-target effects
Translational Status	Preclinical	Preclinical	Preclinical	Approved for aphthous ulcers

### 6.2 Natural products and traditional Chinese medicine formulations

In recent years, a substantial body of research has demonstrated that the therapeutic potential of numerous natural products and traditional Chinese medicine formulations—many of which possess anti-inflammatory and antioxidant activities—for the treatment of diabetes mellitus is largely attributable to their modulation of the cGAS-STING signaling pathway. This provides a rich repository of pharmacologically active compounds for the development of novel therapeutic strategies targeting diabetes mellitus and its associated complications.

Firstly, among commonly used clinical antidiabetic agents, the therapeutic benefits of metformin extend well beyond its classical hypoglycemic effects. Studies have revealed that metformin can suppress excessive activation of the cGAS-STING pathway through multiple mechanisms. For instance, in a Parkinson’s disease model, metformin was shown to reduce mtDNA leakage by activating Mfn2, thereby inhibiting cGAS-STING signaling, delaying astrocyte senescence, and exerting neuroprotective effects (Wang et al., 2024b). Additionally, evidence indicates that its modulation of AMPK activity is also involved in regulating the cGAS-STING pathway during antiviral immune responses (Zhang et al., 2022). Similarly, fenofibrate, a PPARα agonist, in addition to its role in regulating lipid metabolism, suppresses cGAS-STING pathway activation by alleviating mitochondrial damage and reducing the cytosolic release of mtDNA, demonstrating therapeutic potential in ameliorating DR and diabetic dry eye (DDE) (Sun et al., 2025b; Dong et al., 2023). Collectively, beyond their established hypoglycemic or lipid-lowering actions, the regulation of the cGAS-STING pathway by these commonly used clinical drugs offers a novel molecular rationale for their application in the treatment of diabetic complications.

Secondly, a range of natural compounds has also shown significant potential for therapeutic intervention by targeting this pathway. These compounds primarily interfere with cGAS-STING signaling through two main avenues: firstly, by preserving mitochondrial function to prevent the leakage of mtDNA into the cytosol; and secondly, by directly interfering with the activation of cGAS or STING proteins.

#### 6.2.1 Mitochondrial protection and inhibition of mtDNA leakage

In a diabetic periodontitis model, spermidine was found to clear damaged mitochondria and leaked mtDNA by activating mitophagy, thereby inhibiting STING activation and reversing senescence in human periodontal ligament stem cells (Zhou et al., 2025). Salvianolic acid A has been shown to alleviate skin photodamage by reducing mitochondrial damage and inhibiting cGAS-STING activation mediated by mtDNA leakage (Zuo et al., 2024). Luteolin, by activating the Nrf2 and SIRT3/AMPK antioxidant axes, not only improves mitochondrial function but also suppresses the cGAS-STING and Wnt/β-catenin pathways, thereby ameliorating experimental diabetic retinopathy (Wang et al., 2026b). Collectively, these findings underscore that targeting mitochondrial homeostasis and the cGAS-STING signaling axis represents a promising therapeutic strategy for mitigating senescence-related and inflammatory diseases.

#### 6.2.2 Direct intervention in signaling pathways

In an animal model of T2DM, rosavin was observed to downregulate the expression of key signaling molecules such as ZBP1 and STING1, while concurrently activating autophagy, leading to improved insulin resistance and attenuated liver and kidney damage (Ali et al., 2024). Furthermore, compounds such as emodin and baicalin have also been demonstrated to inhibit the cGAS-STING signaling pathway, either directly or indirectly, exerting protective effects in models of diabetic nephropathy and DCM. These studies indicate that natural products finely regulate the cGAS-STING signaling axis through multi-target, multi-pathway mechanisms, offering a wealth of lead compounds for the intervention of related diseases.

Finally, traditional Chinese medicine formulations, functioning as multi-component, multi-target combinations, offer distinct advantages for modulating the complex network of the cGAS-STING pathway. Research indicates that Jiangtang Tiaozhi Formula (JTTZF) significantly suppresses the activation of the cGAS-STING/TBK1/NF-κB pathway in a mouse model of high-fat diet-induced obesity-associated T2DM, thereby improving glycolipid metabolism disorders and hepatic inflammation (Luo et al., 2025). Qihuang Yishen Formula (QHYS) has been shown to mitigate mitochondrial damage and mtDNA leakage by modulating the cGAS/STING pathway, subsequently inhibiting the inflammatory response in renal tubular epithelial cells and slowing the progression of diabetic nephropathy (Guo et al., 2025). Similarly, Tao Hong Si Wu Decoction (TSD) was found to reduce cardiomyocyte pyroptosis and inflammatory injury by inhibiting the cGAS/STING/NLRP3 axis in a DCM model, thereby improving cardiac structure and function (Shen et al., 2025). Collectively, these studies illuminate the modern scientific rationale underlying the therapeutic efficacy of traditional Chinese medicine formulations in treating diabetes mellitus and its complications through the modulation of the cGAS-STING pathway, and they establish a robust foundation for the future discovery of more targeted therapeutic strategies derived from these formulations.

### 6.3 Targeting mitochondrial quality control

Given that mtDNA leakage is a critical upstream event activating the cGAS-STING pathway, restoring mitochondrial function and homeostasis—referred to as mitochondrial quality control—has emerged as a highly promising intervention strategy. In the context of diabetes mellitus and its complications, metabolic stressors such as hyperglycaemia and lipotoxicity can induce excessive mtROS production, imbalances in mitochondrial dynamics (abnormal fusion and fission), and impaired autophagic flux. These events ultimately lead to the accumulation of dysfunctional mitochondria and the release of mtDNA into the cytosol (Ma et al., 2023a; Yan et al., 2022). Therefore, repairing mitochondrial damage through multiple pathways to eliminate the activation signals of cGAS-STING at the source represents a fundamental strategy for reversing downstream inflammatory cascades. Based on this rationale, various intervention strategies targeting mitochondrial quality control have recently demonstrated potential therapeutic value.

#### 6.3.1 Mitochondrial ROS inhibition

Directly reducing mitochondrial reactive oxygen species (mtROS) production is a primary measure to preserve mitochondrial integrity and prevent mtDNA oxidative damage. Fenofibrate, a PPARα agonist, has been shown to alleviate corneal inflammation in diabetic mice by inhibiting ROS production, restoring mitochondrial membrane potential, decreasing mtDNA cytoplasmic leakage, and subsequently suppressing cGAS-STING pathway activation in diabetic dry eye models (Sun et al., 2025b). Similarly, isorhamnetin stabilizes mitochondrial membrane potential, decreases mtROS production, and attenuates diabetic kidney disease by improving mitochondrial homeostasis and inhibiting the cGAS-STING/NLRP3 inflammasome pathway (Zhang et al., 2026). The mitochondria-targeted antioxidant Mito-TEMPO inhibits the activation of cGAS-STING pathway by protecting mitochondrial integrity, thereby alleviating diabetes mellitus-related atrial fibrillation (Meng et al., 2026). Sesamin, anatural lignan, has been found to bind STING with high affinity, inhibit cGAS-STING activation, restore insulin signaling, improve glucose uptake, and enhance mitochondrial respiratory function, demonstrating that high-fat-induced cGAS-STING activation underlies mitochondrial dysfunction, inflammation, and insulin resistance (Xu et al., 2026). Additionally, coenzyme Q10 (CoQ10), a lipophilic antioxidant, can also improve mitochondrial function and attenuate diabetic myocardial ischaemia-reperfusion injury (Xiong et al., 2023). In summary, mitochondria-targeted antioxidant interventions can effectively block the aberrant activation of the cGAS-STING pathway driven by oxidative stress.

#### 6.3.2 TFAM stabilization

Mitochondrial transcription factor A (TFAM) is essential for maintaining mtDNA integrity and packaging. TFAM downregulation leads to abnormal mtDNA packaging, rendering it susceptible to oxidative damage and release into the cytosol, thereby activating the cGAS-STING pathway. Targeting the TFAM–cGAS–STING axis has been proposed as a promising novel strategy for both therapeutic intervention and mechanistic research in diabetic kidney disease (Yun et al., 2026). Restoring TFAM expression or function is considered an effective strategy to inhibit this pathway and reduce inflammation (Yun et al., 2026). For instance, the nuclear receptor FXR can improve mitochondrial function by upregulating TFAM via the ATF4/NRF1 axis, thereby suppressing the activation of the cGAS-STING pathway in diabetic retinopathy (Wang et al., 2025b). These findings establish TFAM as a key molecule for maintaining mtDNA-mitochondrial homeostasis and preventing autoinflammation. The Qihuang Yishen formula has also been shown to ameliorate mitochondrial structural damage, upregulate the expression of TFAM and TOM20, and suppress cGAS/STING pathway activation in DKD models (Guo et al., 2025).

#### 6.3.3 Mitophagy enhancement

Mitophagy specifically eliminates damaged and dysfunctional mitochondria, thereby reducing mtDNA leakage and subsequent cGAS-STING activation. Spermidine, a naturally occurring polyamine, has been shown to clear cytosolic double-stranded DNA of mitochondrial origin by promoting mitophagy, thereby inhibiting STING phosphorylation and downstream signaling and attenuating high glucose-induced senescence in human periodontal ligament stem cells (Zhou et al., 2025). The AhR-FICZ axis represents another critical mitophagy regulatory mechanism. AhR deficiency suppresses mitophagy via impairment of the PINK1/Parkin pathway, leading to the accumulation of damaged mitochondria and subsequent mtDNA leakage, which activates the cGAS-STING-NLRP3 inflammasome axis in diabetic wounds. Activation of AhR by FICZ restores mitophagy, clears damaged mitochondria, prevents mtDNA leakage, and thereby indirectly suppresses cGAS-STING pathway activation (Wang et al., 2025a). Furthermore, the adipokine Metrnl activates autophagy via the LKB1/AMPK/ULK1 signaling axis, promoting the degradation of STING protein, which in turn inhibits the cGAS/STING pathway and ameliorates DCM (Lu et al., 2023). These findings indicate that enhancing mitophagy not only clears damaged organelles but also directly intervenes in the persistent activation of the STING signaling pathway.

#### 6.3.4 Fusion/fission modulation

Mitochondria constantly undergo fusion and fission, and this dynamic balance is essential for their function. Research has revealed that in diabetic myocardial ischaemia-reperfusion injury, a reduction in the mitochondrial fusion protein MFN2 leads to mitochondrial fragmentation, exacerbating mtDNA release and activation of the cGAS-STING pathway (Xiong et al., 2023). Therefore, stabilizing the expression or function of MFN2 helps maintain mitochondrial network integrity. Leptin has been shown to safeguard against hyperglycemia-induced mitochondrial oxidative damage and diabetic cardiomyopathy by modulating the cGAS/STING signaling cascade and Opa1-mediated mitochondrial fusion (Zhang et al., 2025a). Conversely, excessive activation of mitochondrial fission can also cause damage. For example, under high glucose conditions, dynamin-related protein 1 (DRP1)-mediated excessive mitochondrial fission promotes the release of mtDNA into the cytosol via the VDAC1 channel, activating the cGAS-STING pathway and inducing vascular endothelial cell senescence. Using the DRP1 inhibitor Mdivi-1 or the natural product homoplantaginin can block this process and protect vascular endothelial function (Wang et al., 2024a). In diabetic atrial fibrillation, mitochondrial quality control dysregulation—including impaired mitophagy, imbalance in fusion and fission, and reduced mitochondrial biogenesis—has been identified as a key driver of cGAS-STING pathway activation (Meng et al., 2026). These results suggest that maintaining the precise balance between mitochondrial fusion and fission is a critical step in preventing mtDNA leakage and the associated inflammatory response.

#### 6.3.5 NAD^+^ metabolism

NAD^+^ metabolism has emerged as a critical regulator of mitochondrial function and inflammation, and its precursor nicotinamide riboside (NR) has been shown to prevent multiple manifestations of kidney dysfunction in db/db mice with type 2 diabetes—including albuminuria, elevated urinary KIM1 excretion, and pathological changes (Myakala et al., 2023). These protective effects were associated with reduced inflammation, mediated at least in part through inhibition of the cGAS-STING signaling pathway, as corroborated by similar renoprotection observed with a STING antagonist or whole-body STING deletion in diabetic mice. Further mechanistic analysis revealed that NR enhanced SIRT3 activity and improved mitochondrial function, which in turn decreased mitochondrial DNA damage—a key trigger for mitochondrial DNA leakage that activates the cGAS-STING pathway (Myakala et al., 2023). In parallel, the AMPK/SIRT1/PGC-1α axis constitutes a central regulatory network coordinating mitochondrial biogenesis and function, and its activation by NAD^+^ precursors or pharmacological agents has emerged as a promising therapeutic strategy for metabolic diseases (Wang et al., 2026a; Chen et al., 2025c). Collectively, these findings indicate that NR supplementation boosts NAD^+^ metabolism to augment mitochondrial function and attenuate inflammation, thereby slowing the progression of diabetic kidney disease. Notably, hyperglycemia depletes NAD^+^ levels, which consequently suppresses SIRT1 activity and triggers mitochondrial dysfunction along with excessive ROS accumulation (Yen et al., 2024). Therefore, targeting the NAD^+^/SIRT3 axis may represent an effective approach to ameliorate diabetes-associated mitochondrial dysfunction.

#### 6.3.6 MERCs and Ca^2+^ regulation

Mitochondria–endoplasmic reticulum contact sites (MERCs) function as transient signaling platforms that regulate essential cellular functions, including calcium homeostasis, lipid metabolism, and mitochondrial dynamics. Dysregulation of MERCs has been linked to aberrant activation of the cGAS-STING pathway. In adipocytes, high-fat diet leads to structural and functional dysregulation of MERCs, resulting in ER stress and impaired calcium homeostasis, which induces interferon responses centered on STING and triggers insulin resistance. Hoxa5 has been shown to reduce MERC production, alleviate ER stress and calcium over-transport, and affect cGAS-STING-mediated innate immune responses, thereby alleviating insulin resistance and ameliorating metabolic distortions in adipose tissue (Chen et al., 2024). In diabetic retinopathy, high glucose conditions promote excessive Ca^2+^ transfer from the ER to mitochondria via activation of the IP3R1-GRP75-VDAC1 axis (Li et al., 2023). Subsequent mitochondrial calcium overload triggers the opening of the mPTP and the release of mtDNA, thereby activating the cGAS-STING pathway (Li et al., 2023). MERCs may provide a platform for STING to mediate IFN signaling; when mitochondrial dynamics are abnormal, higher ER–mitochondrial coupling and calcium exchange may result in STING residing within the Ca^2+^ microdomains of MERCs, leading to severe ER stress (Chen et al., 2024). Thus, disruptions in MERCs function represent a critical hub linking metabolic stress to mitochondrial inflammation. Targeting key molecules involved in MERCs, such as GRP75, or intervening in calcium transport, may represent promising novel strategies to inhibit the cGAS-STING pathway.

#### 6.3.7 Mitochondrial transfer

Mitochondrial transplantation or transfer represents an emerging therapeutic strategy for restoring mitochondrial function in damaged cells. Apoptotic vesicle membrane-mediated targeted endothelial mitochondrial transplantation has also been explored for diabetic wound healing (Hu et al., 2026). Furthermore, polysaccharide-engineered mitochondria have been shown to reprogram diabetic wound macrophages via targeted mitochondrial transplantation, offering a promising immunometabolic strategy for chronic wound therapy (Yu et al., 2026). However, a critical consideration is that the introduction of allogeneic mtDNA holds the potential to inadvertently activate innate immune-inflammatory cascades via the cGAS-STING axis in recipient cells; if the organelle ruptures in the extracellular space, it will trigger the cGAS-STING pathway, causing massive localized inflammation rather than regeneration. Therefore, optimizing delivery efficiency and ensuring mitochondrial integrity are essential for the safe and effective application of mitochondrial transfer strategies (Table 4).

**TABLE 4:** Natural products and formulations modulating cGAS-STING in diabetes.

Compound /Formulation	Target/Mechanism	Model/Disease	Key effects
Metformin	AMPK activation; Mfn2 upregulation; ALIX degradation; inhibits nuclear egress of chromatin fragments	PD model; DKD; antiviral immunity	Reduced mtDNA leakage, inhibited cGAS-STING, delayed senescence
Fenofibrate	Alleviates mitochondrial damage; reduces cytosolic mtDNA release	DR; DDE; monocytes	Inhibited cGAS-STING activation, attenuated inflammation
Spermidine	Promotes mitophagy; clears mitochondrial-derived dsDNA	Diabetic periodontitis	Reversed PDLSC senescence, blocked STING-TBK1 phosphorylation
Salvianolic acid A	Reduces mitochondrial damage; inhibits mtDNA leakage-mediated cGAS-STING	Skin photodamage	Alleviated skin damage, inhibited cGAS-STING activation
Luteolin	Activates Nrf2 and SIRT3/AMPK; improves mitochondrial function	Experimental DR	Suppressed cGAS-STING and Wnt/β-catenin pathways
Rosavin	Downregulates ZBP1 and STING1; activates autophagy	HFD/STZ-induced T2DM	Improved insulin resistance, alleviated liver/kidney damage
Isorhamnetin	Stabilizes MMP; reduces mtROS; improves mitochondrial homeostasis	DKD	Inhibited cGAS-STING/NLRP3 inflammasome, attenuated DKD
Sesamin	Binds STING with high affinity; inhibits cGAS-STING activation	HFD-induced metabolic dysfunction	Restored insulin signaling, improved glucose uptake, enhanced mitochondrial respiration
Jiangtang Tiaozhi Formula (JTTZF)	Inhibits cGAS-STING/TBK1/NF-κB pathway	HFD-induced obesity-associated T2DM	Improved glycolipid metabolism, hepatic inflammation
Qihuang Yishen Formula (QHYS)	Modulates cGAS/STING pathway; upregulates TFAM and TOM20	DKD	Mitigated mitochondrial damage, inhibited renal tubular inflammation
Tao Hong Si Wu Decoction (TSD)	Inhibits cGAS/STING/NLRP3 axis	DCM	Reduced cardiomyocyte pyroptosis, improved cardiac function
Mito-TEMPO	Scavenges mtROS; protects mitochondrial integrity	Diabetic atrial fibrillation; DCM	Reduced mtDNA leakage, inhibited cGAS-STING, alleviated arrhythmia
Coenzyme Q10 (CoQ10)	Improves mitochondrial function	Diabetic myocardial I/R	Attenuated cGAS-STING activation, reduced injury
Nicotinamide riboside (NR)	Increases NAD^+^, activates SIRT3; improves mitochondrial function	DKD	Reduced mtDNA damage, inhibited cGAS-STING, renoprotection

## 7 Future perspectives

**FIGURE 6:**
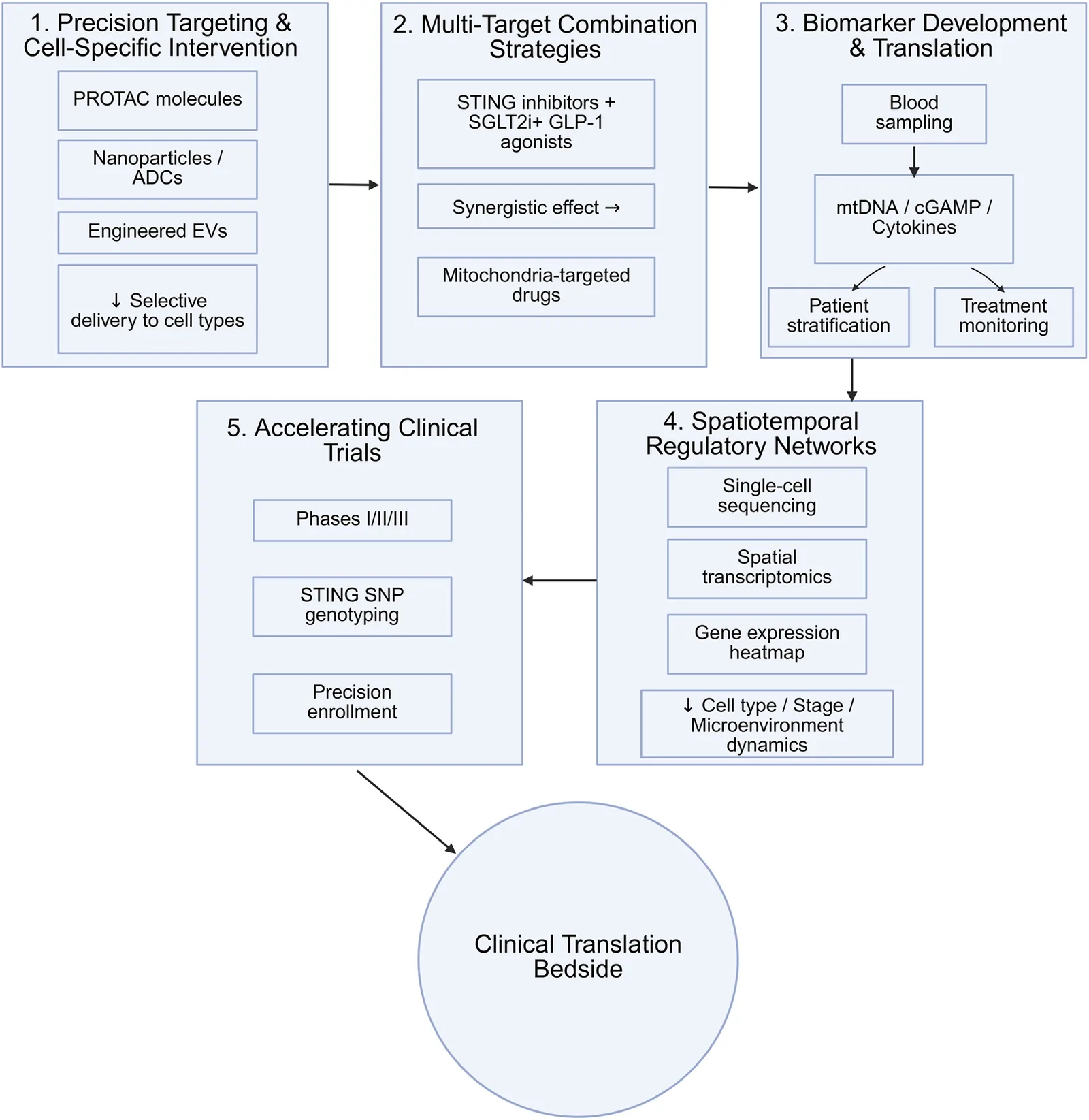
Future directions for clinical translation of cGAS-STING targeting strategies. Proposed future directions include: 1 - Precision targeting using PROTACs, nanoparticles, antibody-drug conjugates, and engineered extracellular vesicles for cell-specific delivery; 2 - Multi-target combination strategies (combining STING inhibitors with SGLT2 inhibitors, GLP-1 receptor agonists, or mitochondria-targeted agents); 3 - Biomarker development (blood-based mtDNA, cGAMP, and cytokine panels for patient stratification and treatment monitoring); 4 - Spatiotemporal mapping via single-cell sequencing and spatial transcriptomics to capture cell-type-, disease stage-, and microenvironment-specific dynamics; 5 - Accelerated clinical trials incorporating STING single nucleotide polymorphism genotyping for precision patient enrollment. Created in BioRender. (2026) https://BioRender.com/58sh5w6.

In summary, the cGAS-STING signaling pathway plays a central pathological role in the onset and progression of diabetes mellitus and its associated complications. Given that research in this field remains in a phase of rapid development, future investigations should focus on the following areas to facilitate the translation of fundamental discoveries into clinical applications (Figure 6).

### 7.1 Precision targeting and cell-specific intervention

Although various small-molecule inhibitors targeting cGAS or STING (e.g., RU.521, C-176, H-151) have been developed and demonstrated therapeutic potential in preclinical models (Ma et al., 2023a; Xiong et al., 2023; Mitrofanova et al., 2022), their potential off-target effects and the long-term risks associated with systemic inhibition, such as increased susceptibility to infections, warrant careful evaluation. Consequently, future research should prioritize the development of more selective allosteric inhibitors or Proteolysis Targeting Chimeras (PROTACs) to achieve precise modulation of this pathway (Cai et al., 2025). More importantly, given that the cGAS-STING pathway may exert distinct pathological roles in different cell types (e.g., immune cells versus parenchymal cells) (Mitrofanova et al., 2022; Liu et al., 2023), the development of cell-type-specific drug delivery systems—such as antibody-drug conjugates, nanoparticles, or engineered extracellular vesicles—will facilitate precision intervention. This strategy aims to enhance therapeutic efficacy while minimizing systemic side effects (Deng et al., 2024; He et al., 2025). Therefore, an in-depth understanding of cell-specific regulatory mechanisms and the development of corresponding targeted delivery strategies are crucial for achieving precise intervention of the cGAS-STING pathway.

In diabetic kidney disease, STING activation in podocytes is clearly pathogenic, as it drives proteinuria and podocyte loss (Mitrofanova et al., 2022). By contrast, cGAS in pancreatic β-cells may exert a partially protective role by promoting β-cell proliferation through a STING-independent, CEBPβ-dependent mechanism, indicating that components of the cGAS-STING pathway can exhibit cell-type-specific and even opposing functions (Cai et al., 2024). This context-dependent signaling is further illustrated by the PGE2-EP receptor system, in which the same ligand elicits divergent effects depending on receptor subtype and cellular milieu, thereby highlighting the need for precision targeting (Yang et al., 2026). Such functional duality underscores the critical importance of cell-type-resolved analyses to differentiate pathogenic from compensatory activation, which is essential for designing interventions that selectively suppress detrimental pathway activity while preserving any beneficial functions. Moreover, macrophage polarization adds yet another layer of complexity: cGAS-STING activation promotes M1 pro-inflammatory polarization, a clearly pathogenic process, whereas its role in tissue-resident macrophage homeostasis remains context-dependent and less straightforward.

### 7.2 Multi-target combination therapeutic strategies

The pathogenesis of diabetes mellitus is complex, and monotherapy targeting a single pathway is often insufficient to halt disease progression. Consequently, this multi-target combination therapy paradigm is increasingly recognized as a necessary strategy for complex diseases, precisely because it directly addresses the interconnected nature of pathogenic signaling networks (Gong et al., 2026; Li et al., 2026b). Given the interplay between the cGAS-STING pathway and various pathological processes—including metabolic stress, inflammation, fibrosis, and cell death—combination therapy strategies hold promise for achieving synergistic efficacy. For instance, preclinical studies have suggested that combining STING inhibitors with existing anti-diabetic drugs (such as SGLT2 inhibitors or GLP-1 receptor agonists) or with anti-fibrotic agents may more comprehensively ameliorate diabetic nephropathy or cardiomyopathy (Wang et al., 2024b; Pang et al., 2025). Moreover, strategies targeting upstream mitochondrial dysfunction (e.g., using Mito-TEMPO) (Meng et al., 2026) or modulating autophagy (e.g., through Metrnl or spermidine) (Zhou et al., 2025; Lu et al., 2023) have also demonstrated synergistic effects when combined with cGAS-STING pathway inhibition. Therefore, future research should systematically evaluate the synergistic mechanisms, optimal combinations, and safety profiles of these combination therapies, so as to provide superior therapeutic regimens for clinical practice.

### 7.3 Development and clinical translation of biomarkers

To translate findings on the cGAS-STING pathway into clinical practice, there is an urgent need to develop non-invasive or minimally invasive biomarkers that reflect the activity of this pathway. Circulating levels of mtDNA, cGAMP concentrations, and specific inflammatory cytokine profiles (e.g., interferon, IL-1β) have been shown to correlate with pathway activation status (An et al., 2023; Khedr et al., 2024; Yang et al., 2025). However, the specificity, sensitivity, and dynamic association of these markers with disease stage and treatment response require validation through large-scale, prospective clinical cohort studies. In-depth exploration and validation of these circulatory biomarkers will facilitate patient stratification, early diagnosis, therapeutic monitoring, and the development of individualized treatment strategies, thereby effectively bridging the gap between basic research and clinical practice.

A key question for biomarker development is which circulating biomarkers best reflect tissue STING activation. Emerging evidence suggests that plasma cGAMP, a direct product of cGAS enzymatic activity, may serve as a more specific indicator of pathway activation than downstream cytokines (Zhang et al., 2025b). Circulating cell-free mtDNA has also been proposed as a surrogate marker for mitochondrial damage and subsequent cGAS-STING activation (Zhao et al., 2022c). However, the specificity of these markers for tissue-level STING activation remains uncertain, as they may also reflect systemic inflammation from other sources. Future studies should prioritize the identification of tissue-specific STING activation signatures—potentially through the detection of cell-type-specific exosomal cargo or the development of activity-based probes that can be detected in biofluids—to enable precise monitoring of pathway activity in target organs without invasive biopsies.

### 7.4 In-depth elucidation of spatiotemporally specific regulatory networks

The activation and function of the cGAS-STING pathway exhibit high spatiotemporal specificity. Advanced technologies such as single-cell sequencing and spatial transcriptomics enable the analysis of the pathway’s dynamic changes across different cell types, disease stages, and tissue microenvironments at single-cell resolution (Du et al., 2024; Huang et al., 2025b). For instance, studies have revealed that STING is predominantly enriched in endothelial cells in diabetic retinopathy (Qin et al., 2026), whereas its activation occurs earliest in podocytes in diabetic nephropathy (Mitrofanova et al., 2022). Therefore, future research should leverage these technologies to construct high-resolution spatiotemporal maps of the pathway in diabetes mellitus and its complications, elucidating its complex roles in disease initiation, progression, and resolution. This will provide a robust theoretical foundation for selecting optimal intervention timings and targets.

### 7.5 Accelerating clinical translation and precision medicine applications

Two additional translational questions merit attention. First, can local STING inhibition avoid systemic immunosuppression? Preclinical studies have demonstrated the feasibility of localized delivery strategies. These approaches aim to achieve therapeutic efficacy at the disease site while minimizing systemic exposure and the associated risks of immunosuppression. However, the translation of these local delivery strategies to clinical practice requires further optimization of delivery efficiency, retention time, and safety profiles. Second, which current antidiabetic drugs modulate cGAS-STING indirectly? Metformin has emerged as a key example. Metformin modulates cGAS-STING signaling and promotes AMPK-mediated KLF2 activation (Alanazi et al., 2026). GLP-1 receptor agonists have also been suggested to modulate key inflammatory pathways including cGAS-STING (He et al., 2024b). Understanding these indirect modulatory effects may inform combination strategies and drug repurposing opportunities for cGAS-STING-targeted therapy in diabetic complications.

Advancing safe and effective cGAS or STING inhibitors into clinical trials represents an urgent priority in the field. Although small-molecule inhibitors have shown promise in animal models, their pharmacokinetic properties, long-term safety, and efficacy in the complex context of diabetes mellitus must be rigorously validated through clinical trials (Ma et al., 2023a). Furthermore, given that differences in patient genetic background, such as STING single nucleotide polymorphisms (SNPs), may influence disease susceptibility and treatment response (Hamann et al., 2020), future clinical trial designs should incorporate such factors and explore precision treatment models based on biomarkers. Particularly for diabetic complications with poor responses to current therapies, such as DFU (He et al., 2024a; Pang et al., 2025) and diabetic nephropathy (Khedr et al., 2024), therapies targeting the cGAS-STING pathway hold promise as a novel and breakthrough treatment strategy, offering new hope for these challenging conditions.

## 8 Conclusion

In summary, by synthesizing a large volume of recent discoveries alongside established knowledge, this review provides an updated and critical framework for understanding the cGAS-STING pathway in diabetes and its complications. Diabetes mellitus and its complications represent a major global public health challenge, underpinned pathologically by chronic, low-grade metabolic inflammation. In recent years, the cGAS-STING signaling pathway has emerged as a critical hub linking cellular stress to sterile inflammation, making its mechanistic role in diabetes mellitus and its complications a research Frontier. This review systematically elucidates the mechanisms of pathway activation under metabolic stress, its pathological involvement in various complications, and evaluates the prospects and challenges of targeting this pathway therapeutically.

Regarding the mechanisms of activation, metabolic stressors such as hyperglycemia and lipotoxicity engage the cGAS-STING pathway through three primary routes. First, mitochondrial dysfunction provokes the leakage of mitochondrial DNA (mtDNA) into the cytosol, where it serves as a damage-associated molecular pattern (DAMP) to ignite the pathway—a process exacerbated by excessive reactive oxygen species production, aberrant mitochondrial fusion/fission dynamics, impaired autophagy, and the modulatory action of TFAM. Second, hyperglycemia-induced nuclear DNA damage and subsequent micronucleus formation also trigger this pathway, operating in parallel with the mitochondrial route. Furthermore, endoplasmic reticulum (ER) stress can independently precipitate mitochondrial dysfunction and mtDNA release, a phenomenon mediated by calcium overload at mitochondria–ER contact sites (MERCs); notably, the ER stress response and its unfolded protein response (UPR) branches constitute a conserved pathogenic hub that, when dysregulated, amplifies mitochondrial injury and inflammation across diverse disease contexts (Chen et al., 2026b). Collectively, these interconnected stress pathways converge on cGAS-STING activation, underscoring the central role of organellar crosstalk in metabolic inflammation and suggesting that targeting this network may offer novel therapeutic opportunities for chronic metabolic diseases.

In terms of pathological roles, aberrant activation of the cGAS-STING pathway is extensively involved in major diabetic complications. In DCM, the pathway mediates cardiomyocyte pyroptosis, pathological hypertrophy, and cardiac dysfunction. In DKD, its activation drives podocyte injury, renal tubular epithelial cell inflammation and pyroptosis, and promotes renal fibrosis via the TFAM-cGAS-STING axis, with regulation by epigenetic modifications such as RNA m6A methylation. In diabetic retinopathy, the pathway is activated in Müller cells and endothelial cells, mediating neuroinflammation, vascular leakage, and cellular senescence, while the intercellular transfer of cGAMP amplifies microglia-mediated neuroinflammation. In DFU, pathway activation leads to an imbalance in macrophage M1/M2 polarization, premature senescence of fibroblasts and keratinocytes, and forms a vicious cycle of inflammation through a mechanical-metabolic “double-hit” mechanism. Additionally, this pathway is implicated in various other complications, including macrovascular disease, atrial fibrillation, lung injury, periodontitis, dry eye disease, and neurocognitive impairment.

The pathway is also deeply involved in the core pathological processes of diabetes mellitus itself. In adipose tissue, its activation exacerbates insulin resistance. In pancreatic β-cells, it mediates metabolic stress- and DNA damage-induced β-cell senescence, apoptosis, and dysfunction, playing a critical role in both T1DM and T2DM.

Significant progress has been made in therapeutic strategies targeting this pathway. Small molecule inhibitors, such as STING inhibitors (e.g., C-176, H-151), the cGAS inhibitor RU.521, and the TBK1 inhibitor amlexanox, have demonstrated protective effects in various preclinical models. Furthermore, natural products (e.g., spermidine, salvianolic acid A, salidroside) and traditional Chinese medicine formulations (e.g., Jiangtang Tiaozhi Fang, Qihuang Yishen Fang) have shown therapeutic potential by modulating this pathway. Given that mtDNA leakage is a key upstream event in pathway activation, targeting mitochondrial quality control—such as improving mitochondrial function, regulating dynamics and autophagy—represents an effective strategy to block inflammatory signals at their source.

Despite its promising potential, the clinical translation of targeting this pathway faces several challenges. These include the cell-type and tissue specificity of the pathway, the potential risk of systemic immunosuppression and infection, functional redundancy with other DNA sensors, a lack of specific biomarkers, and difficulties in targeted drug delivery. Future research should focus on developing cell-specific intervention strategies, exploring multi-target combination therapies, establishing reliable biomarker panels, and utilizing single-cell and spatial omics technologies to map the spatiotemporal dynamics of the pathway. Such efforts will be crucial to propel the clinical translation of cGAS-STING pathway-targeted therapies for diabetes mellitus and its complications, offering novel breakthrough solutions for these challenging diseases.

## Glossary

cGAS: cyclic GMP-AMP synthase
STING: stimulator of interferon genes
DM: diabetes mellitus
T1DM: type 1 diabetes mellitus
T2DM: type 2 diabetes mellitus
DKD: diabetic kidney disease
DR: diabetic retinopathy
DCM: diabetic cardiomyopathy
DFU: diabetic foot ulcer
mtDNA: mitochondrial DNA
dsDNA: double-stranded DNA
DAMPs: damage-associated molecular patterns
ROS: reactive oxygen species
mPTP: mitochondrial permeability transition pore
TLRs: Toll-like receptors
NLRs: NOD-like receptors
cGAMP: 2′3′-cyclic GMP-AMP
TBK1: TANK-binding kinase 1
IRF3: interferon regulatory factor 3
IFN-I: type I interferons
NF-κB: nuclear factor-kappa B
MFN2: mitofusin 2
DRP1: dynamin-related protein 1
VDAC1: voltage-dependent anion channel 1
TFAM: mitochondrial transcription factor A
ER: endoplasmic reticulum
MERCs: mitochondria-endoplasmic reticulum contact sites
TECs: tubular epithelial cells
ESRD: end-stage renal disease
m6A: N6-methyladenosine
iBRB: inner blood-retinal barrier
AhR: aryl hydrocarbon receptor
FICZ: 6-formylindolo[3,2-b]carbazole
NLRP3: NOD-, LRR- and pyrin domain-containing protein 3
SASP: senescence-associated secretory phenotype
MQC: mitochondrial quality control
HIIT: high-intensity interval training
PROTACs: Proteolysis Targeting Chimeras
PPARα: peroxisome proliferator-activated receptor α
SGLT2: sodium-glucose cotransporter 2
GLP-1: glucagon-like peptide-1
AMPK: AMP-activated protein kinase
MLKL: mixed lineage kinase domain-like protein
ZBP1: Z-DNA-binding protein 1
AIM2: absent in melanoma 2
IFI16: interferon-gamma inducible protein 16
DDX41: DEAD-box helicase 41
Opa1: optic atrophy 1
LKB1: liver kinase B1
ULK1: UNC-51-like kinase 1
ISG15: interferon-stimulated gene 15
PINK1/Parkin: PINK1/Parkin
FXR: farnesoid X nuclear receptor
ATF4: activating transcription factor 4
NRF1: nuclear respiratory factor 1
mtROS: mitochondrial reactive oxygen species
SNPs: single nucleotide polymorphisms
HFD: high-fat diet
STZ: streptozotocin
NAFLD: non-alcoholic fatty liver disease
CVD: cardiovascular disease
CKD: chronic kidney disease
IL-1β: interleukin-1β
IL-6: interleukin-6
IL-18: interleukin-18
TNF-α: tumor necrosis factor-α
TGF-β1: transforming growth factor-β1
VEGF: vascular endothelial growth factor
MCP-1: monocyte chemoattractant protein-1
ICAM-1: intercellular adhesion molecule-1
VCAM-1: vascular cell adhesion molecule-1
CCL2: C-C motif chemokine ligand 2
CCR2: C-C motif chemokine receptor 2
AGEs: advanced glycation end-products
RAGE: receptor for advanced glycation end-products
iNOS: inducible nitric oxide synthase
NO: nitric oxide
MDA: malondialdehyde
SOD: superoxide dismutase
Nrf2: nuclear factor erythroid 2-related factor 2
Keap1: Kelch-like ECH-associated protein 1
SIRT1: sirtuin 1
SIRT3: sirtuin 3
mTOR: mammalian target of rapamycin
STAT3: signal transducer and activator of transcription 3
STAT6: signal transducer and activator of transcription 6
YAP: Yes-associated protein
TAZ: transcriptional coactivator with PDZ-binding motif
EMT: epithelial-mesenchymal transition
MDSCs: myeloid-derived suppressor cells
PD-L1: programmed death-ligand 1
PD-1: programmed cell death protein 1
ADCs: antibody-drug conjugates
NPs: nanoparticles
GelMA: gelatin methacryloyl
PDLSCs: human periodontal ligament stem cells
IHC: immunohistochemistry
IF: immunofluorescence
TEM: transmission electron microscopy
H&E: hematoxylin and eosin
ELISA: enzyme-linked immunosorbent assay
RNA-seq: RNA sequencing
WB: Western blotting
LC-MS/MS: liquid chromatography-tandem mass spectrometry
MD: molecular docking
SAVI: STING-associated vasculopathy with onset in infancy
